# Current status and future prospects of brain–computer interfaces in the field of neurological disease rehabilitation

**DOI:** 10.3389/fresc.2026.1666530

**Published:** 2026-02-10

**Authors:** Yu Luo, Xiaohu Liu, Miaomiao Yang

**Affiliations:** College of Nursing, Dali University, Dali, Yunnan, China

**Keywords:** brain-computer interface, neuroplasticity, neurorehabilitation, non-invasive/minimally invasive treatment (NIMT), stroke

## Abstract

Neurological disorders represent a significant category of diseases that profoundly affect human health, accounting for the second leading cause of global mortality. This group of conditions includes stroke, multiple sclerosis (MS), amyotrophic lateral sclerosis (ALS), spinal cord injury, Parkinson's disease, and cerebral palsy, among others. These disorders are highly susceptible to sequelae and profoundly impact individuals’ daily lives. In this context, Brain-Computer Interface (BCI) technology has demonstrated considerable potential in the domain of neurorehabilitation, although numerous challenges remain. The manuscript provides a comprehensive review of recent advancements in research and clinical applications, highlighting current limitations and outlining future directions. It elucidates the applicability and constraints of Brain-Computer Interface (BCI) technology across various diseases and patient populations. To facilitate insights across different conditions, comparative tables are presented, aligning BCI strategies with therapeutic targets, outcomes, advantages, limitations, and existing evidence gaps. The scope extends beyond motor restoration to include under-explored domains, such as neuropathic pain, with a focus on real-world translation, including home and community feasibility and the distinction between assistive and rehabilitative applications. The review distills overarching limitations within the field, such as small sample sizes, protocol heterogeneity, and limited longitudinal evidence, while synthesizing the most recent studies. An actionable research and development roadmap is proposed to guide next-generation BCI rehabilitation, incorporating individualized cortical-network simulators, self-architecting decoders, adaptive therapy approaches akin to game seasons, and proprioceptive “write-back” mechanisms via peripheral interfaces. Moreover, the review reveals significant research focal points and critical issues that warrant further investigation in the context of neurological rehabilitation utilizing BCI technology.

## 1 Introduction

In rehabilitation medicine, rehabilitation of the sequelae of neurologic disorders has always been a complex, challenging and particularly important area. Neurologic disorders including stroke, multiple sclerosis (MS), amyotrophic lateral sclerosis (ALS), spinal cord injury (SCI), Parkinson's disease (PD), and cerebral palsy are the major causative factors for severe motor dysfunction and disability. Neurological diseases (excluding stroke, brain and neurological cancers, and infectious neurological diseases) are the second leading cause of death globally, accounting for 16.8% (1). Stroke has become the third leading cause of death and disability globally (2). Recent epidemiological studies demonstrate a significant increase in stroke prevalence, paralleled by rising mortality rates (3, 4). A large number of stroke survivors still have severe hand function disorders, limited ankle control and abnormal gait in the chronic stage, which restricts their daily life and social participation (5, 6). A considerable proportion of stroke survivors also experience long-lasting cognitive impairments, particularly in attention and executive function, which are highly prevalent in post-stroke cognitive impairment (7). However, conventional stroke rehabilitation has historically focused on motor recovery, and hidden cognitive deficits are often under-recognized and insufficiently targeted by standard rehabilitation programs (8). A soft pneumatic muscle actuator specifically developed for elbow joint rehabilitation has been designed, simulated, and systematically evaluated, demonstrating precise control of elbow flexion and extension and offering improved flexibility, comfort, and customization compared with traditional rigid exoskeletons (9). Distinct from the soft pneumatic actuator described above, a wearable surface electromyography (sEMG) sensor system combined with an adaptive machine learning algorithm can accurately recognize various daily-life hand gestures, providing the possibility for long-term, continuous assessment of hand function and human–computer interaction (10). The ankle and elbow rehabilitation equipment based on soft pneumatic muscles adopts a compliant and muscle-like driving method to achieve safer, more comfortable and customizable upper and lower limb rehabilitation training, and is gradually moving towards home and community scenarios (11). The training using robotic hands combined with virtual reality (VR) not only improves the motor function of the affected hand in patients with chronic stroke, but also significantly enhances their cognitive abilities such as attention and execution (4). The rope-driven flexible hand rehabilitation robot, combined with EEG monitoring, has demonstrated that it can activate the activities of relevant brain regions during training, suggesting that this technology has the potential to promote cortical reorganization by stimulating active participation (12). These studies highlight a growing effort to develop adjunctive technologies that can complement conventional physical and occupational therapy and help deliver higher-intensity, task-specific, and more sustainable rehabilitation for stroke survivors. As a result, new technologies such as wearable robots, soft exoskeletons, and VR have emerged. These systems mainly rely on peripheral signals (electromyography, kinematics), while BCI directly utilizes brain signals, which is expected to more accurately capture “remaining neural networks+neural plasticity”, thereby providing supplementary or even unique value in stroke rehabilitation. Beyond stroke, other major neurological disorders—such as multiple sclerosis (MS), amyotrophic lateral sclerosis (ALS), spinal cord injury (SCI), Parkinson's disease (PD) and cerebral palsy (CP)—also pose profound challenges to neurorehabilitation and long-term functional independence (13). Across these conditions, inflammatory, degenerative or developmental damage to the central nervous system leads to chronic motor, sensory and cognitive impairments that often persist despite multidisciplinary standard-of-care rehabilitation (14–16). In this broader context, BCIs are being explored as a cross-cutting strategy: primarily as motor-rehabilitation adjuncts in MS and SCI, communication and control interfaces in ALS, adaptive neuromodulation tools in PD, and engagement- and learning-enhancing paradigms in children with CP (17–21). Collectively, these examples illustrate that BCIs provide a disease-independent framework that directly links cortical activity to feedback-based interventions, offering a unified yet customizable platform for enhancing neuroplasticity and functional recovery across diverse neurological disorders (22).

Considering the global population growth and aging trends, the incidence of these diseases is expected to rise further (23). For the patient, these diseases often cause a loss of motor ability, a significant reduction in the ability to perform activities of daily living, or even complete dependence on others for ADL(24). This dependency not only limits the patient's independence and freedom, but it can also lead to a damaged sense of self-esteem and even cause depression and social isolation (25). For families, taking care of these patients requires a huge investment of time, energy and financial resources, which may increase the pressure on families and even affect the physical and mental health and career development of family members (26, 27). At the social level, the high morbidity and disability rates of these diseases place a heavy burden on the public health system. As the number of patients continues to grow, there is a corresponding rise in the demand for specialized medical personnel, rehabilitation services, long-term care facilities, and assistive technologies (28). Furthermore, the inability of numerous patients to continue working can significantly affect social productivity and economic activity (29). These factors collectively render the treatment and rehabilitation of neurological disorders not only a medical challenge but also a significant social and economic concern. Therefore, there is an urgent need for effective rehabilitation strategies to improve dysfunction in patients with these neurological disorders and brain injuries and to increase the independence of affected individuals.

The advent of BCI technology has brought new hope to the field of neurorehabilitation. Conventional rehab improves function through therapist-guided, task-specific practice that depends on residual movement and behavioral performance(30, 31). In contrast, BCI trains the brain by decoding motor intention and delivering intention-contingent feedback/assistance (VR/robot/FES), enabling training even without overt movement (32, 33). BCI time-locks feedback/assistance to detected motor-intent brain patterns, so Hebbian/ spike-timing–dependent plasticity (STDP) pairing is precise (34); and it operantly conditions target neural biomarkers (while discouraging compensations) (35, 36)—levels of specificity and timing standard task practice can't match. Traditional training is more like “you open the door first, and only afterwards does someone tell you whether it was right or wrong,” whereas BCI makes the door open at the very moment your “neural finger” presses the doorbell. This tight temporal contingency between intention-related brain activity and feedback is a key reason why BCIs can more efficiently drive experience-dependent brain reorganization. Although the pathology and pathophysiological mechanisms of these disorders vary, they all share the potential for neuroplasticity - the ability of the brain to reorganize and form new connections after learning and injury (37). This inherent plasticity is central to neurological disease recovery and forms the foundation for therapeutic interventions. BCI technology is based on the principle of neuroplasticity, restoring or compensating for lost neurological function, thereby facilitating recovery from disease and improving the overall quality of life for patients with neurological disorders (22, 38).

BCI technology is a novel and promising approach that opens up a new channel for translating brain intent into actual action by accurately capturing brain activity and decoding neural intent, translating brain signals in real time, and converting the neural signals into commands for controlling external devices (39). It is especially suitable for patients with motor dysfunction due to stroke or other neurological disorders (40). Depending on how it is implemented, a BCI can act either as an assistive interface or as a rehabilitative tool. In the rehabilitative setting, its core advantage lies in the ability to bypass damaged efferent pathways and link intention-related electrophysiological signals directly to congruent sensory, visual, or proprioceptive feedback from external devices (e.g., robotic movement or FES-induced contraction). By allowing patients to repeatedly attempt movements while the corresponding cortical populations are active and immediately receive contingent feedback, BCI-based training creates a closed-loop, intention-contingent learning environment that is thought to strengthen spared sensorimotor circuits and promote experience-dependent neuroplasticity, which over time can translate into measurable improvements in motor performance (41). With the deepening of research, BCI technology has progressed by enhancing signal-processing accuracy through deep-learning-based EEG decoding (42, 43), developing portable and user-friendly systems for daily rehabilitation (44, 45), and integrating multimodal feedback such as virtual-reality- or FES-coupled training that improves functional outcomes (40). As a means of assisting in rehabilitation therapy, brain-computer interface (BCI) technology not only provides a non-invasive or minimally invasive treatment option (24), but also introduces innovative treatment strategies beyond conventional therapy, such as closed-loop BCI+robotics/FES or VR-BCI paradigms (46, 47). Additionally, BCI technology provides real-time, intention-contingent feedback, allowing users to immediately perceive how their brain activity influences external devices. Beyond its role in temporally precise pairing between intention and feedback, this immediacy can strengthen the user's sense of agency and enhance engagement and motivation, which are important for sustaining high-dose, repetitive training in neurorehabilitation (48), and allows for personalized adjustment of training difficulty based on individual performance. Consequently, BCI technology plays a crucial role in promoting neurological rehabilitation and improving the quality of life for individuals with functional disabilities. BCI technology has shown significant potential for realizing highly personalized treatment plan, for example by employing closed-loop neurofeedback systems that adjust in real time to each patient's brain-state and by tailoring feedback devices and protocols to individual neural-activation patterns (49). This potential is demonstrated by the ability of BCI systems to recognize and utilize the unique brain activation patterns of each patient, allowing for the personalization of BCI training strategies (50). At the same time, adaptive BCI systems provide online assistance to patients who have difficulty manipulating traditional BCI systems, thus expanding the boundaries of the application of BCI technology in rehabilitation therapy (51). In addition, the introduction of deep learning models has enabled the prediction of rehabilitation outcomes after BCI training, providing guidance for individualized protocols in the early stages of clinical rehabilitation (52). The continuous development and application of these technologies have collectively accelerated the progress of BCI technology in the field of neurorehabilitation therapy.

**Figure 1:**
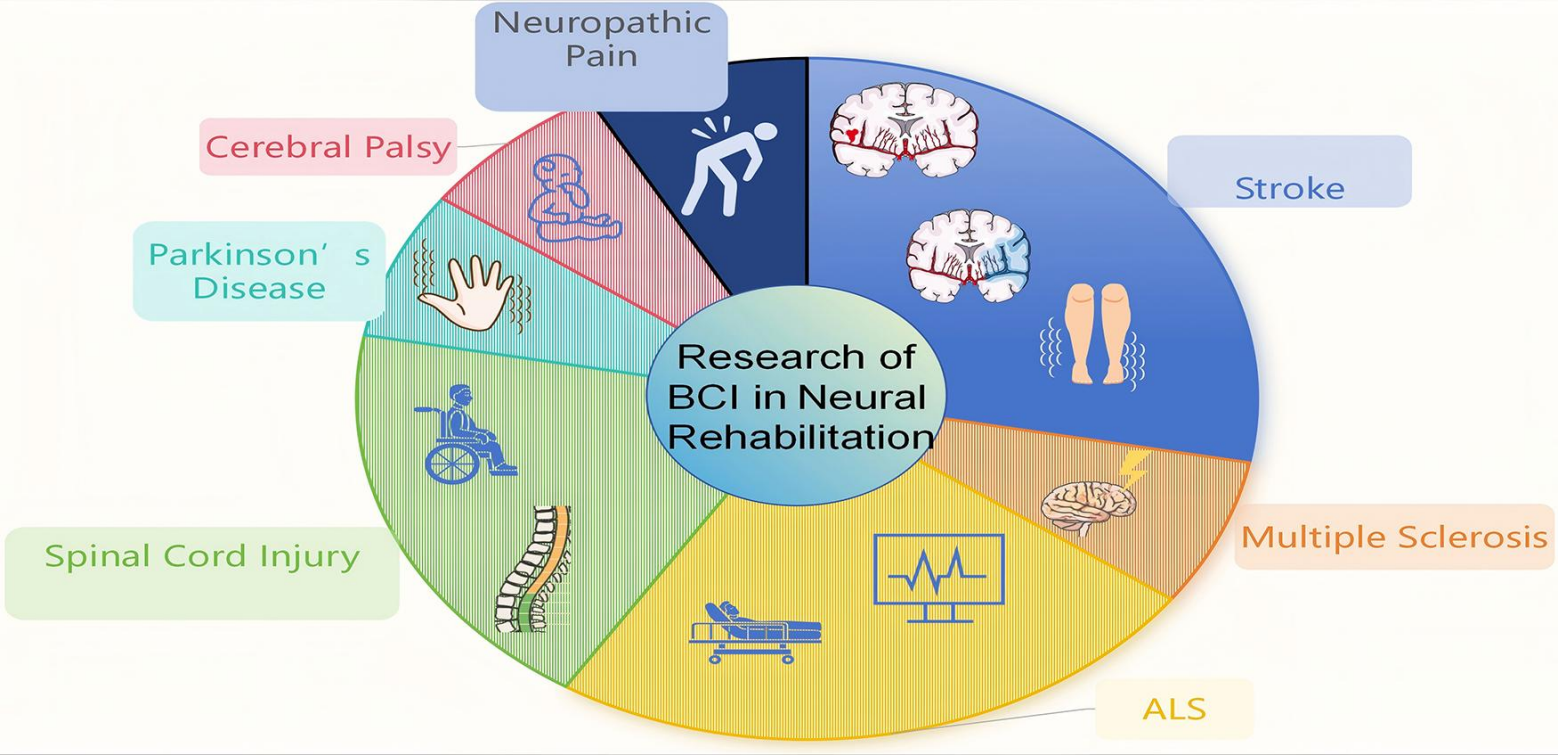
Distribution of BCI applications across neurological conditions. The figure qualitatively summarizes the relative focus of brain–computer interface (BCI) research across major neurological disorders discussed in this review. Stroke and amyotrophic lateral sclerosis (ALS) are most frequently investigated, followed by spinal cord injury. Research on Parkinson's disease, neuropathic pain, multiple sclerosis, and cerebral palsy is comparatively limited but gradually expanding. The proportions are intended to illustrate relative research emphasis rather than quantitatively represent the entire field.

We explore the use and research advancements of BCI technology in rehabilitating neurological disorders like stroke, multiple sclerosis (MS), amyotrophic lateral sclerosis (ALS), spinal cord injury (SCI), Parkinson's disease (PD) and cerebral palsy. As illustrated in Figure 1, stroke and ALS represent the predominant focus of current BCI research, followed by spinal cord injury, whereas studies in other disorders remain relatively limited but are steadily increasing. As an innovative tool that translates brain activity to control external devices, BCI offers new treatment and rehabilitation strategies. The paper aims to highlight BCI's potential in this area and guide future research, anticipating further breakthroughs and improved outcomes for patients. The current status and key limitations across major neurological conditions are summarized in Table 1.

**Table 1:** Overview of the current status and shortcomings of brain-computer interfaces in the field of neurorehabilitation.

Disease	Research progress	Research limitations	References
Stroke	Improved motor function recovery using BCI. Development of evaluation tools and neuroplasticity studies.	Lack of personalized rehabilitation strategies. Insufficient long-term effect research.	(33), (103–111) (112–114)
Multiple Sclerosis	Feasibility and classification accuracy of BCI explored. Application of BCI with functional electrical stimulation (FES).	Limited sample size and unclear generalizability. Insufficient evaluation of quality of life.	(161), (164–166)
ALS	Enhanced communication through adaptive BCI systems. Advances in speech decoding and motor intention detection.	High training demands for late-stage patients. Limited focus on psychological and emotional impacts.	(193–199)
Spinal Cord Injury	Potential for restoring motor functions with BCI.	Individual variability and long-term adaptability issues. Limited understanding of physiological feedback.	(228–232)
Parkinson's Disease	Significant improvement in motor function using BCI.		(258) (266, 267)
Cerebral Palsy	Initial exploration of BCI application in rehabilitation for perinatal stroke cases.	Limited studies on children with cerebral palsy. Need for further clinical investigations.	(297–299)
Neuropathic Pain	Non-pharmacological pain relief methods proposed using BCI.	Lack of studies on individual variability in treatment effects.	(221, 326) (332–334)

## 2 The mechanism of brain–computer interfaces in neurorehabilitation

The neuroscience foundation is the theoretical support of BCI technology. Neuroscience research has revealed the electromagnetic activities of the brain that accompany the generation of nerve impulses, which are rhythmically and spatially specific (53). BCI technology utilizes sensors to capture and augment these specific neurophysiological signals to interpret the brain's intentions (54).

BCI can be used as a tool to stimulate and enhance neuroplasticity. Neuroplasticity refers to the ability of the brain to reorganize and repair itself in response to experience, learning, environmental changes, or injury. It is the foundation for brain development, learning, memory, and adaptive behavior (55, 56). This ability is manifested on multiple levels, including synaptic plasticity - the ability of synaptic connections to change in strength and efficiency (57). Neuroplasticity is also manifested as experience-dependent plasticity (58), whereby the structure and function of the brain changes in response to an individual's experience, e.g., learning a new skill increases the density of gray matter in a particular brain region. In addition, structural plasticity involves changes in the physical structure of neurons, synapses, and neural networks (59), while functional plasticity refers to the variability in the functional expertise of brain regions, especially after brain damage, when other regions may take over the functions of the damaged region (60, 61). Neuroplasticity is the basis of neurorehabilitation, where the brain is able to adapt to lost function by reorganizing itself (62). The processes of learning and memory involve the strengthening and reorganization of neural networks, which is a direct manifestation of neuroplasticity (63). Environments that provide rich stimuli can promote neuroplasticity (64, 65), thereby enhancing learning and memory. At the synaptic level, abundant stimulation induces NMDA receptor-dependent long-term potentiation (LTP) and regulates the glutamate and other neurotransmitter systems, thereby enhancing synaptic transmission efficiency (66). At the structural level, the abundant stimuli promote adult hippocampal neurogenesis through the BDNF-mTOR/Wnt signaling pathway, and drive an increase in dendritic spine density and axonal collateral sprouting to form new neural functional circuits (67). Based on the principle of neural plasticity, when two neurons are simultaneously subjected to frequent stimulation, the synaptic connection between them will be strengthened (68). This is of vital importance for associative learning and the development of new neural pathways. The frequently used neural circuits become stronger and more efficient due to the increase in synaptic weights, while the less frequently used circuits deteriorate. The selective enhancement of synaptic connections between neurons provides the foundation for joint learning and the stable solidification of new neural pathways (69, 70). The brain-computer interface (BCI) can create a high-dimensional, dynamic and behaviorally-related “rich environment” through real-time, closed-loop, and multimodal sensory-motor stimulation. By targeting and regulating the above multi-scale plasticity mechanisms, it can accelerate functional reorganization and behavioral recovery. The study of neuroplasticity is important for understanding how the brain adapts to change, how it recovers from injury, and how to optimize therapeutic strategies (71).

BCI technology stimulates neuroplasticity in the brain by utilizing appropriate feedback mechanisms to awaken dormant neurons and prompt them to form specific neurotransmission pathways. Based on the principle of neuroplasticity, this activation enhances cortical network connectivity for the purpose of neural remodeling (72). BCI technology restores damaged neural networks by decoding goal-directed intentions and providing beneficial feedback loops. This feedback mechanism promotes neuroplasticity and helps patients restore or compensate for lost neural function. Repeated neurofeedback or closed-loop training with BCI systems over extended periods can drive activity-dependent modulation within cortical networks, either through endogenous engagement of neural circuits or through externally triggered peripheral or cortical stimulation, thereby promoting neuronal recruitment, strengthening synaptic connectivity, enhancing neuroplasticity, and facilitating neurological recovery (73).

**Figure 2:**
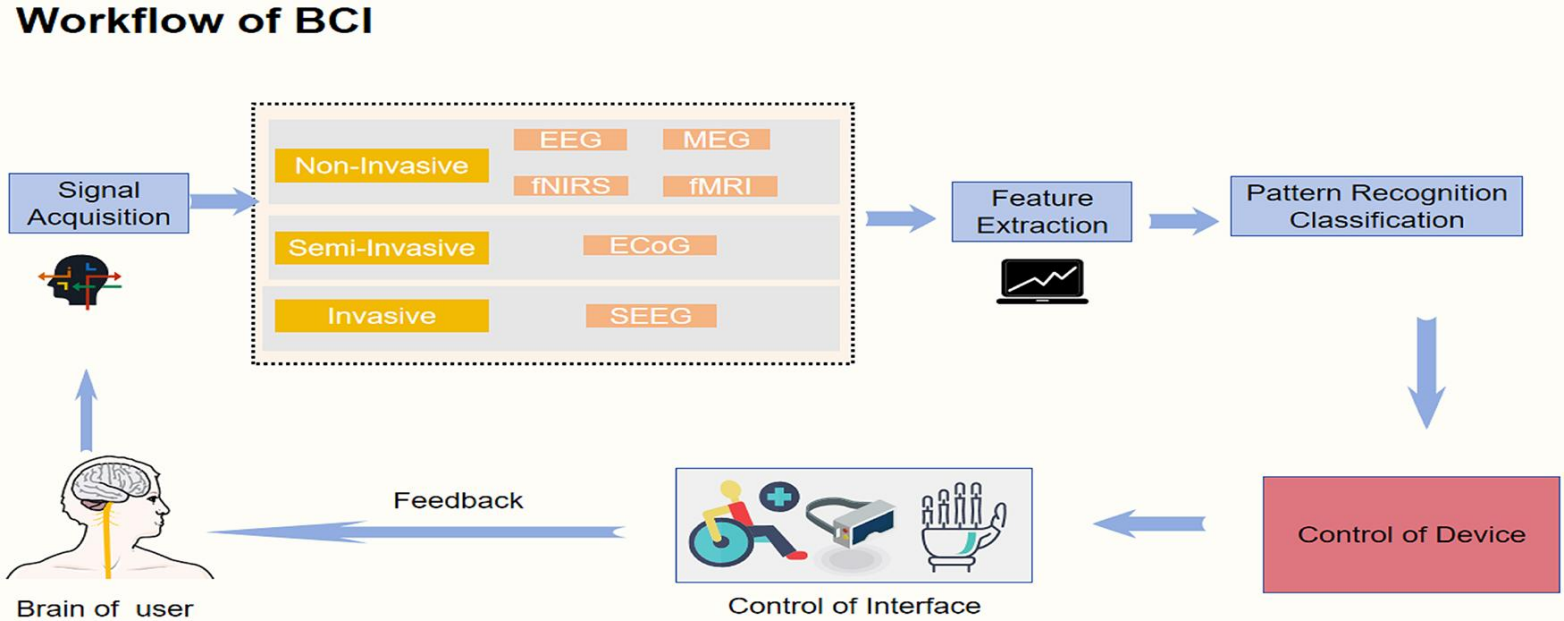
Workflow of a brain–computer interface (BCI) system. The diagram illustrates the general workflow of a BCI system, comprising signal acquisition, signal processing, feature extraction, pattern recognition/classification, and device control, followed by a feedback pathway that returns information to the user. Neural signals are acquired through non-invasive (e.g., EEG, MEG, fNIRS, fMRI), semi-invasive (e.g., ECoG), or invasive (e.g., SEEG) methods. After feature extraction and decoding, the system translates neural activity into control commands for external interfaces or assistive devices such as robotic arms, wheelchairs, or functional electrical stimulators. The feedback pathway allows users to adjust or refine their control based on sensory or visual cues, forming a self-regulated interaction loop that supports adaptive performance and user learning.

At the heart of BCI technology is the capacity to record and interpret brain activity and translate it into machine-readable command (74). Neurons communicate via electrical impulses (75), and these signals can be detected and recorded using specialized neurophysiological equipment (76). In a typical BCI system, electrical activity is captured by sensors placed on or in the brain and by related biosignal sensors, such as electroencephalography (EEG), electromyography (EMG) and electrooculography (EOG), which measure brain- and muscle- or eye-related signals (77). The raw data are then preprocessed—filtered and amplified—to reduce noise and retain informative components. From these cleaned signals, features related to the user's intentions are extracted, for example through statistical and spectral analyses. Finally, a classifier maps these features onto specific control commands that drive external devices (78). The general workflow of a BCI system, from neural signal acquisition through preprocessing and feature extraction to external device control, is illustrated in Figure 2. A cross-condition comparison of BCI strategies and rehabilitation targets is provided in Table 2.

**Table 2:** Comparison of BCI systems applied in different neurological rehabilitation domains (sections 3.1–3.7).

Neurological Condition	BCI Strategy/System Type	Targeted Rehabilitation Function	Advantages	Disadvantages/Limitations	Main Experimental Outcomes
Stroke	MI-BCI+FES/VR/soft robotic glove; hybrid BCI (MI + SSVEP)	Upper-limb, hand,gait rehabilitation	Enhances neuroplasticity; supports motor relearning; provides multisensory feedback	Small sample sizes; little inter-patient variability; signal instability	Significant improvement in FMA-UE, ARAT, gait speed; improved kinesthetic perception
Multiple Sclerosis (MS)	EEG-based source-level/discrete BCI; BCI-FES for gait	Gait and motor function recovery	Demonstrates feasibility and non-invasive; can improve gait speed	Small cohorts; limited control groups; lack of long-term data	Improved gait speed and event-related desynchronization latency
Amyotrophic Lateral Sclerosis (ALS)	Invasive (ECoG) and non-invasive BCIs for speech/text; P300 + AR systems	Communication restoration	Enables text or speech output; stable long-term use; improves autonomy	Surgical risks; reduced accuracy in complete LIS; high training demand	Stable decoding accuracy > 97%; up to 32 wpm communication rate; long-term home use proven
Spinal Cord Injury (SCI)	Intracortical BCI; Brain-Spine Interface (BSI); BCI-FES; MI-BCI	Motor reconstruction, grasping, walking	Restores voluntary motion; enables natural walking; portable home systems	Individual variability; long-term adaptability not verified	Re-established walking and grasping; improved upper-limb control; strong user satisfaction
Parkinson's Disease (PD)	EEG+EMG fusion; MI-BCI; multimodal fNIRS-EEG	Tremor and rigidity reduction; motion detection	Detects motor intent; supports tremor mitigation	Requires complex preprocessing; small sample sizes	High classification accuracy (88–89%); reduction of rigidity and tremor
Cerebral Palsy (CP)	EEG-based BCI; user-centered home BCI	Motor and cognitive engagement in children	Demonstrates feasibility for children; promotes independence	Limited sample; long-term efficacy unknown	Comparable task accuracy to controls; successful home implementation
Neuropathic Pain	BCI+VR + TENS; BCI-music therapy	Pain modulation and emotional regulation	Provides real-time pain monitoring; non-pharmacological	High individual variability; effect dependent on preference	50% reduction in pain intensity (NPSI); improved emotional regulation

## 3 Generalized BCI implementations in neurorehabilitation

### 3.1 Common BCI paradigms and signal types

Brain–computer interfaces can be broadly categorized according to the type of neural signal they exploit and the paradigm used to elicit control. In neurorehabilitation, most systems rely on non-invasive EEG-based BCIs that decode modulation of sensorimotor rhythms during motor imagery (MI-BCI) (79). In motor imagination (MI) paradigms, patients are instructed to imagine specific movements (e.g., grasping, ankle dorsiflexion or stepping), which induce event-related desynchronization and synchronization (ERD/ERS) in µ and *β* bands over sensorimotor cortex (80). Changes in these rhythms can be transformed into continuous or discrete control signals that trigger feedback, such as FES-induced contraction or robotic movement (81).

Other commonly used paradigms include P300-based BCIs, which detect event-related potentials elicited by infrequent target stimuli in oddball tasks, and steady-state visual evoked potential (SSVEP) BCIs, which decode frequency-specific responses to flickering visual stimuli (82). P300 and SSVEP systems are particularly suited for communication and selection interfaces, for example in patients with ALS or severe motor impairment who cannot reliably perform motor imagery (83). Hybrid BCIs combine two or more paradigms (e.g., MI plus P300 or SSVEP) or integrate EEG with other biosignals such as EMG or eye movements, in order to improve robustness, allow mode switching and adapt to disease-specific limitations (46). Across the neurological disorders reviewed in this article, these paradigms constitute the main “building blocks” from which disease-specific BCI applications are constructed.

### 3.2 Typical signal processing and feedback modalities

Despite differences in disease and paradigm, most rehabilitation-oriented BCIs share a similar processing pipeline (84). Neural or related biosignals are first acquired using non-invasive sensors such as EEG, EMG or EOG, or in some experimental settings with invasive electrodes. The raw signals are then band-pass filtered, artifact-reduced and, when appropriate, spatially filtered to enhance task-relevant activity and suppress noise (46, 85, 86). From the preprocessed data, features that represent the user's intention are extracted, for example band power changes in predefined frequency bands (ERD/ERS), temporal characteristics of event-related potentials, or spectral peaks at stimulation frequencies in SSVEP paradigms. Machine-learning classifiers or regression models then map these features onto discrete commands or continuous control signals (87, 88).

The final stage of the BCI loop is feedback and actuation. In neurorehabilitation, decoded intentions are most often used to trigger or modulate assistive and therapeutic modalities, including FES to specific muscles, robotic or exoskeleton-assisted movement, virtual or augmented reality environments, and, in some studies, adaptive neuromodulation (47–90). Crucially, feedback is delivered in close temporal proximity to the detected intention-related activity, creating a closed sensorimotor loop that links cortical activation to congruent visual, proprioceptive and tactile consequences (91). This temporally precise, intention-contingent feedback is thought to support Hebbian and spike-timing–dependent plasticity in spared circuits and to enhance the user's sense of agency and engagement, thereby providing a common mechanistic rationale for BCI-based rehabilitation across different neurological disorders (40, 92).

## 4 Research progress of BCI technology in the field of neurorehabilitation

### 4.1 Stroke

#### 4.1.1 Disease presentation and standard of care

Stroke-related movement disorders and functional limitations substantially reduce patients’ quality of life (93), yet conventional rehabilitation methods remain limited in their ability to promote meaningful functional recovery in severely paralyzed patients (94). Post-stroke rehabilitation is organized around specialized stroke units and an interdisciplinary team (physiatry, PT/OT/SLT, nursing, psychology, social work) with coordinated transitions from acute care to inpatient rehabilitation and then to community or early-supported discharge (ESD) for eligible patients (typically mild–moderate strokes. Services aim to maintain continuity of therapy intensity across settings and to deliver goal-directed, task-specific practice (30). Rehabilitation standard of care (SoC) for stroke is team-based and stage-wise. Rehabilitation typically progresses from acute care to inpatient rehabilitation and then to community or early supported discharge (ESD) once patients are medically stable (usually within 24–48 h). Current guidelines recommend delivering high-intensity, task-specific, feedback-rich practice (approximately ≥3 h/day, ≥ 5 days/week when feasible) (95). For the upper limb, SoC includes repetitive task practice, constraint-induced movement therapy (when eligible), and mental practice or mirror therapy, with EMG- or intention-triggered FES and robotic devices commonly used as dose amplifiers (30, 96, 97). For gait, SoC emphasizes task-specific walking training (overground or treadmill) at moderate-to-high intensity, with ankle–foot orthoses or peroneal-nerve FES added as needed for foot drop (31). Manage focal spasticity with botulinum toxin A + therapy/splintin; address prevention, pain, dysphagia, mood/cognition (30). The general prognosis of stroke is highly heterogeneous and influenced by multiple interrelated factors, including the initial severity of neurological deficits, corticospinal tract integrity, lesion location and size, timing of intervention, and the patient's neuroplastic potential (98); nevertheless, guideline-concordant, dose-adequate, task-specific rehabilitation improves independence and participation (99). Even after completing standard rehabilitation, large numbers of stroke survivors worldwide continue to live with persistent functional deficits (100). Given constrained healthcare resources and marked regional disparities, traditional programs alone struggle to deliver adequate coverage and intensity. There is therefore a need for scalable, effective adjuncts that can be layered onto standard care to further enhance recovery (e.g., intention-contingent FES/robotics, telerehabilitation, and BCI).

#### 4.1.2 Role of neuroplasticity in the rehabilitation process

**Figure 3:**
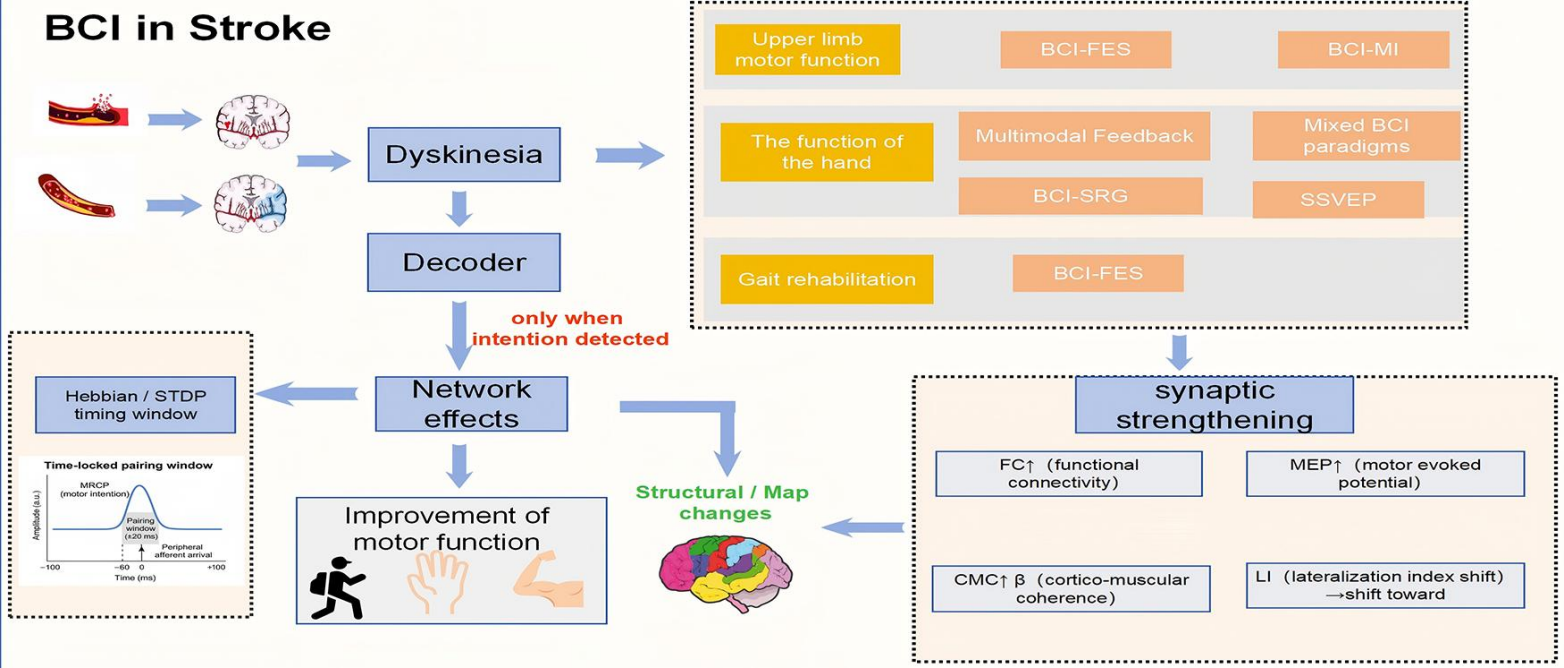
Mechanism of brain–computer interface (BCI)–based motor rehabilitation after stroke. Schemic or hemorrhagic stroke causes disruption of motor pathways, leading to dyskinesia and limb dysfunction. Motor-imagery–based BCI (BCI-MI) systems decode cortical activity associated with the patient's motor intention and use it to trigger time-locked peripheral functional electrical stimulation (FES), forming a closed-loop sensorimotor circuit. This synchronous activation of cortical and peripheral pathways induces Hebbian and spike-timing–dependent plasticity, strengthening corticomuscular connections and promoting motor recovery. Additional paradigms—such as steady-state visual evoked potential (SSVEP) BCIs, sensorimotor rhythm–robotic glove (BCI-SRG) systems, and hybrid multimodal feedback approaches—extend BCI applications to fine hand movements and gait rehabilitation. Together, these mechanisms enhance neuroplasticity and facilitate the restoration of upper-limb and gait function in stroke survivors.

Neuroplasticity—the brain's capacity to reorganize structure and function—underpins recovery after stroke. Following focal injury, spared cortico-subcortical networks resolve diaschisis, unmask latent synapses, and form new connections; with appropriately dosed, task-specific practice, these changes are shaped by Hebbian and spike-timing–dependent plasticity to strengthen task-relevant pathways and suppress inefficient compensations. Intensive, repetitive, feedback-rich training recalibrates interhemispheric excitation–inhibition balance, remaps sensorimotor representations, and enhances corticomuscular coherence, changes that track improvements on impairment and activity scales (101). Put simply, through appropriately dosed, task-specific, feedback-rich, and intention-timed training, spared networks are reactivated and reconnected; interhemispheric balance is recalibrated, sensorimotor maps are remapped, and corticomuscular coherence is strengthened—culminating in measurable gains on clinical scales and in everyday function. Given the principle of neuroplasticity, BCI technology offers an innovative solution to enhance neuroplasticity in stroke patients by directly decoding brain signals to drive external devices, as illustrated in Figure 3. Its potential lies in enabling personalized, closed-loop rehabilitation training to optimize neurological reconstruction. Currently, the application of BCI in stroke rehabilitation has become a hot research area. In routine stroke care, assistive technologies (AFOs/canes, peroneal-nerve FES for foot drop, EMG-triggered FES, and robot-assisted practice) improve safety and dose but generally do not guarantee intention-contingent timing (30). BCI's differentiator is that neural intent itself triggers the assistance/feedback, which may reduce compensatory strategies and better satisfy Hebbian/STDP timing rules during training (102). Representative protocols include MI-BCI combined with FES/VR 30 min/day, ≥ 5 days/week for 1 month, and 3–4 sessions/week for 3–4 weeks, which have yielded larger gains on the Fugl-Meyer Assessment for the Upper Extremity (FMA-UE) and the Action Research Arm Test (ARAT) than dose-matched conventional therapy in reported trials; gait-oriented BCI-FES delivered 3×/week for 4–5 weeks has improved walking speed and posture control. In stroke, BCIs can enhance quality of life (QoL) by turning intention into immediate, task-relevant assistance that drives functional gains which matter for daily living. Because assistance/feedback is triggered by neural intent, BCIs may curb compensatory strategies and better satisfy timing rules for plasticity than dose-matched conventional tools, supporting more efficient practice toward independence and participation. This is reflected by upper-limb improvements on FMA-UE/ARAT after MI-BCI–based programs, ADL-oriented BCI–soft-glove training that fosters sustained functional use/kinesthetic perception, and gait-oriented BCI-FES that can increase walking speed—each a pathway to better self-care and community mobility from a lower QoL baseline in post-stroke disability.

#### 4.1.3 BCI-based rehabilitation in stroke: evidence and outcomes

Most stroke patients have motor deficits (103). Several clinical studies have evaluated BCI-based interventions as adjuncts to conventional post-stroke motor rehabilitation. In a multicenter randomized controlled trial, Wang et al. (33) compared motor-imagery BCI plus standard therapy with standard therapy alone and found significantly greater improvements in Fugl–Meyer Assessment for Upper Extremity (FMA-UE; 0–66 points, higher scores indicate better upper-limb function) in the BCI group after four weeks of training. In a subacute cohort with severe upper-limb impairment, Brunner et al. (104) reported that patients receiving non-invasive MI-BCI–triggered functional electrical stimulation (FES) 3–4 times per week for 3–4 weeks were more likely to achieve clinically meaningful upper-limb gains than those receiving conventional training. In a single-arm exoskeleton-based BCI study, Bhagat et al. (105) delivered 12 sessions of intention-driven elbow training and observed significant improvements in FMA and Action Research Arm Test (ARAT) scores relative to stable baseline values. Collectively, these studies suggest that BCI-controlled FES and robotics are promising adjuncts for upper-limb motor recovery after stroke.

In exploring the application of BCI technology in hand rehabilitation for stroke, several studies have combined BCIs with soft robotic gloves. In this context, “soft” robotic gloves refer to wearable hand exoskeletons made from compliant, back-drivable structures that are lightweight and deformable, allowing safer physical interaction with the paretic hand and more comfortable, natural grasp patterns during ADL-oriented training. For example, Cheng et al. (106) investigated a BCI-based soft robotic glove (BCI-SRG) in chronic stroke, comparing motor-imagery–driven glove activation with glove-assisted training without BCI control under matched session durations over six weeks. In the BCI-SRG group, motor imagery was used to trigger glove-assisted movements during activity-of-daily-living (ADL) tasks. All participants reported vivid movement sensations in the paretic upper limb during training, and in a subset of patients this kinesthetic sensation persisted beyond the intervention period. These findings suggest that, in chronic stroke, coupling BCI with a soft, compliant glove may strengthen the link between movement intention and sensory–motor feedback in meaningful ADL contexts, potentially enhancing engagement, kinesthetic awareness and the effectiveness of hand rehabilitation. In addition, Guo et al. (107) used a steady-state visual evoked potential (SSVEP)–based BCI to detect user intention and trigger a soft robotic glove during 10 one-hour training sessions; by providing a more reliable and straightforward command channel, their system reduced early-stage “failed attempts” and user frustration, improving the feasibility and user experience of glove-based rehabilitation. Zhang et al. (108) developed a hybrid BCI that combines motor imagery with high-frequency SSVEP and couples the decoded intention to a soft robotic glove. By replacing static visual cues with movement-related visual stimulation, the system elicited stronger and more reliable motor-imagery signals, allowing the glove to open and close the hand more consistently in response to user intention. This stability is essential for delivering high-quality, intention-driven repetitions, which form the basis of effective neuroplastic motor rehabilitation. Shao et al. (109) further integrated BCI with a hand rehabilitation system that provides visual, auditory and haptic feedback in a virtual reality (VR) environment, creating an immersive, multisensory context for training. This multimodal feedback is designed to strengthen bidirectional sensory–motor coupling and increase engagement, potentially enhancing the effectiveness and adherence of hand rehabilitation in chronic stroke.

Research into BCI technology has also extended to gait rehabilitation after stroke. Biswas et al. (110) compared a BCI-triggered functional electrical stimulation (BCI-FES) program delivered three times per week for four weeks with conventional physical therapy and reported greater improvements in gait speed in the BCI-FES group, with no major safety issues. Chung et al. (111) applied a similar non-invasive BCI-FES paradigm targeting ankle dorsiflexion on the more affected leg for 30 min per session, three times per week over five weeks, and found beneficial effects on postural control and gait performance in individuals with chronic hemiparesis. Both studies were conducted in small samples, and their findings will need confirmation in larger, longer-term trials.

Most BCI research in stroke targets motor deficits, but an emerging body of work is beginning to explore non-motor domains such as cognition, mood and multisensory feedback. Mane et al. (112) outlined how BCI systems could be used not only to drive motor training but also to monitor and modulate cognitive workload and affective state, arguing for more holistic BCI designs that jointly address motor, cognitive and emotional rehabilitation needs. In the multimodal neurofeedback space, Rieke et al. (113) developed a BCI framework that integrates real-time fMRI and near-infrared spectroscopy to provide feedback on brain activation during wrist-extension training, illustrating how richer neural feedback might help patients learn to engage target networks more consistently. Sánchez-Cuesta et al. (114) further demonstrated the feasibility of combining immersive BCI–VR environments with established neuromodulation techniques, showing that pairing intention-driven control with engaging, multisensory feedback can enhance patient motivation and may amplify the effects of conventional neurorehabilitation protocols. Together, these early studies suggest that BCI in stroke could eventually extend beyond pure motor control to support cognitive, emotional and multisensory aspects of recovery. Currently, the research hotspots of BCI technology based on stroke rehabilitation focus on studying the effects of BCI technology on motor function recovery after stroke, including upper limb, lower limb, and hand functions; exploring the effects and advantages of BCI technology compared with traditional rehabilitation methods; improvement and optimization of the BCI system; clinical translation and utility of BCI techniques and development of new assessment tools and methods to monitor the effects of BCI training.

#### 4.1.4 Mechanistic and clinical implications in stroke

Stroke is a highly heterogeneous disorder, and the mechanisms through which brain–computer interface (BCI) interventions harness neuroplasticity differ across stroke subtypes, lesion sites, and recovery phases. Hemorrhagic and ischemic strokes engage distinct pathophysiological cascades—ischemic injury triggers excitotoxic and inflammatory responses leading to focal disconnection, while hemorrhagic lesions induce diffuse edema and secondary cortical inhibition—both of which differentially shape the extent and time course of functional reorganization (115). Even among ischemic strokes, different lesion patterns affect distinct motor networks (116). Consequently, the neural circuits available for rehabilitation differ across patients.

The location and extent of brain injury determine whether patients can successfully perform motor imagery tasks and whether brain-computer interfaces can “read out” meaningful information from their brain signals (117). When the primary motor cortex—particularly the precentral “hand-knob” region—is severely damaged, classical contralateral event-related desynchronization (ERD) patterns become unreliable (118). When patients imagine performing an action, the corresponding area of the brain fails to generate normal electrical signal patterns, and thus the BCI is unable to recognize them. In such cases, neuroimaging and BCI studies have demonstrated compensatory activation of ipsilateral premotor cortex (PMC), supplementary motor area (SMA), and posterior parietal cortex (PPC), which can serve as alternative control sites for MI-based training (40, 72). Adaptive decoders of BCI that dynamically identify and track these compensatory patterns enable the system to capitalize on residual cortical plasticity even when canonical hand-motor areas are lost (119).

As outlined in Section 3.2, closed-loop BCIs that time-lock motor intention to immediate sensory or proprioceptive feedback are particularly suited to exploit stroke-related neuroplasticity. From a neuroplasticity perspective, the timing of BCI intervention in stroke is critical. In the subacute phase, surviving perilesional tissue shows heightened excitability and synaptic receptivity, creating an optimal “plasticity window” in which pairing motor intention with contingent sensory feedback or functional electrical stimulation (FES) is most likely to drive reorganization (120). In the chronic phase, BCIs can still be beneficial, but meaningful gains may require higher-intensity, longer-duration and often multimodal stimulation to re-engage partially dormant networks, because the physiological substrate for large-scale re-learning is reduced compared with earlier stages (121).

Existing BCI-based stroke rehabilitation studies vary greatly in training frequency, duration, and total dose, making it unclear whether therapeutic outcomes depend more on total practice time or on individual responsiveness. Because post-stroke neural plasticity, signal stability, and patient endurance vary substantially across individuals, rigid rehabilitation timelines are physiologically unrealistic (122). Future work should therefore develop adaptive scheduling frameworks in which session length, intensity, and feedback modality are tailored to each patient's evolving neural and behavioral state. A key challenge will be determining how to balance total training dose with flexibility and personalization to maximize recovery efficiency.

Consequently, not all BCI-based interventions achieve consistent success across studies. Some studies have found that the effects of BCI are not obvious or unstable (102). One of the reasons for this is that these studies did not achieve “real-time closed-loop feedback” during the training process (119). That is to say, the intention signals sent by the brain and the feedback provided by the machine (such as moving the arm or the screen moving) are not synchronized, resulting in neurons not being activated at the “same time”, and thus unable to effectively form or strengthen new connections. The second reason is that these studies began training after the “critical period of natural neural reorganization” following a stroke had ended. The first few weeks to several months after a stroke is the “easiest window period for neural reorganization”, and after this period, neural plasticity decreases, and the training effect is naturally not as significant as early intervention (123). In summary, the effect of BCI is not “one-size-fits-all” and cannot simply be regarded as “effective for all stroke patients”. Its efficacy depends on: the location and type of brain injury (lesion characteristics), the integrity of the remaining brain network (residual connectivity), and the timing of intervention (neurophysiological timing).

In addition to neurophysiological mechanisms, the psychological state of the patient plays an important role in post-stroke recovery. Emotional distress, depression, and low motivation are common after stroke and are known to negatively influence adherence, engagement, and overall rehabilitation outcomes under standard care (124). Conversely, active participation, self-efficacy, and positive emotional states enhance motor learning and neuroplasticity. Because BCI-based rehabilitation relies heavily on sustained attention and voluntary modulation of brain activity, fluctuations in motivation or emotional status can directly affect signal quality and training effectiveness (125). Therefore, integrating psychological assessment and motivational feedback into BCI protocols may be essential for achieving consistent therapeutic results. From a clinical perspective, these findings support personalized BCI-augmented rehabilitation, in which BCI-related parameters (e.g., target cortical regions and feedback modality) and rehabilitation dose parameters (e.g., session duration and frequency) are tailored to each patient's neural status. This patient-specific tuning of both the interface and the training dose aligns with the broader goal of precision neurorehabilitation.

#### 4.1.5 Limitations and future directions

In the domain of stroke rehabilitation, the investigation of BCI technology has achieved substantial advancements. However, several challenges and research gaps remain. For instance, long-term effects and neuroplasticity have not been sufficiently explored. Research on multimodal data fusion technology is still in its nascent stages, necessitating further technical refinement and development. The accuracy and stability of BCI systems also encounter significant obstacles. Due to brain injuries, the dynamic characteristics of EEG signals are altered, making it challenging to acquire a sufficient amount of high-quality EEG data. While BCI systems have demonstrated potential in laboratory settings, their application in real-world clinical environments is more complex than anticipated. The engagement and enjoyment of BCI training in existing studies require enhancement to improve patient participation and focus during training sessions. Some studies inadequately account for patients’ sensory functions, despite the critical role of sensory feedback in motor function recovery. Additionally, the small sample sizes in many studies limit the reliability and generalizability of the findings. Given the considerable variability among patients in terms of neurological injury patterns, rehabilitation requirements, and recovery trajectories, BCI systems must be capable of recognizing these differences and adapting to the unique needs of each individual. Future studies should develop adaptive BCI systems that not only recalibrate decoders between sessions but also support within-session (online) adaptation of signal decoding to accommodate neural variability and adjust feedback or task difficulty based on the patient's recovery stage. Specifically, such adaptive systems would update decoding parameters (e.g., feature scaling, thresholds or classifier weights) across days and within sessions to account for fluctuations in neural activity caused by fatigue, changes in arousal, electrode displacement or ongoing neural reorganization. In practice, this could include brief calibration at the beginning of each therapy session to “re-train” the decoder for that day, combined with online adaptation during use as signal properties drift. At the same time, the system could adjust task difficulty and feedback intensity according to the patient's clinical progress, for instance, by gradually reducing external assistance from functional electrical stimulation (FES) or virtual reality (VR) feedback as voluntary motor control improves. Ultimately, such systems could help “wean” patients off BCI dependence, transforming the device from a compensatory aid into a catalyst for sustained cortical reorganization. Moreover, as highlighted earlier, effective personalized rehabilitation programs must consider not only the physiological state of patients but also psychological dimensions—such as emotional status, motivation, and engagement—which critically influence adherence and the efficacy of both conventional and BCI-based therapies. While initial studies have begun to investigate the potential of BCI technology for enhancing cognition and emotion in stroke patients, the number of studies in this field remains limited, necessitating further exploration by researchers.

In non-invasive EEG-based BCIs for stroke rehabilitation, many studies report classification accuracies around 60%–80%, especially when real-time feedback is provided (89) Although information transfer rates (ITRs) and command latencies are rarely reported for rehabilitation tasks, where ITR denotes the amount of information a user can transmit through the BCI per unit time (bits/s), communication-oriented BCIs using similar EEG paradigms have achieved information transfer rates of up to approximately 5 bits/s in laboratory settings (e.g., high-speed speller systems) (126). These figures highlight the growing performance of BCI systems, yet they also underscore that current methods remain below the ideal thresholds for seamless daily-clinical use. While such performance is adequate for proof-of-concept demonstrations, it remains insufficient for seamless real-time interaction and clinical translation. Although precise benchmarks have not been formally defined, accuracies close to 90%, sub-second response times (typically <300 ms), and reliable performance across repeated sessions are often cited as practical goals for clinical-grade BCI systems (127). Current limitations arise from the nonstationary nature of EEG signals, inter-subject variability, and signal contamination from artifacts such as muscle activity or eye movement, which degrade the robustness of feature extraction and classification (128, 129). Furthermore, conventional decoding algorithms often rely on static calibration models that fail to adapt to neural changes during rehabilitation (130). Therefore, future research could build on emerging hybrid BCI work by integrating EEG with other physiological signals such as electromyography (EMG) or heart rate variability (HRV). Hybrid EEG–EMG systems in stroke and motor control tasks have already shown that combining cortical and muscular signals can improve intention detection accuracy, robustness to artifacts and early detection of movement onset compared with EEG alone (131, 132). Similarly, several studies suggest that HRV reflects workload and impending loss of sensorimotor rhythm control in motor imagery BCIs, indicating that it may serve as a useful adjunct marker of engagement and control quality (133). Leveraging such multimodal neural and peripheral indicators may allow future BCIs to capture motor intention with higher precision and responsiveness and to adapt assistance in a more natural, clinically usable way for stroke patients. Moreover, considering individual variability in lesion distribution and recovery stage—often represented by different Brunnstrom levels—future studies should develop personalized BCI training protocols that align with each patient's residual neural resources and reorganization pattern. Even when the primary motor cortex is severely affected, previous research has shown that compensatory or non-motor paradigm —such as the premotor cortex (PMC), supplementary motor area (SMA), and posterior parietal cortex (PPC)—can still provide effective neural control signals, underscoring the feasibility of tailoring BCI engagement to the patient's functional network rather than to a single cortical area (40, 72).At the same time, advancing cost-effective, portable, and user-friendly BCI systems will be critical for broader clinical translation. Reducing hardware costs, simplifying calibration, and enhancing ease of use will determine how effectively these technologies can move from laboratory prototypes to everyday rehabilitation practice.

### 4.2 Multiple sclerosis (MS)

#### 4.2.1 Disease presentation and standard of care

Multiple sclerosis (MS) is a chronic autoimmune disease that affects the central nervous system, particularly the brain and spinal cord, and is characterized by an inflammatory response that destroys the protective myelin sheath surrounding nerve cells (134). In areas affected by MS, nerve signaling is slowed or blocked, and in turn neurological symptoms occur, leading to a reduced quality of life and disability (135). The disease is the most common non-traumatic disabling disease affecting young people (136) and its incidence and prevalence is increasing year by year and becoming a global disease (137). In multiple sclerosis (MS), sensorimotor involvement is highly heterogeneous because lesion topography (periventricular and juxtacortical cortex, brainstem/cerebellum, and spinal cord) and lesion biology (active demyelination vs. chronic axonal loss) vary across individuals and over time. Demyelination causes conduction block and temporal dispersion, producing fluctuating weakness, slowed motor output, and pronounced fatigability, whereas axonal transection and gray-matter atrophy lead to more persistent deficits (138, 139). Spinal cord lesions yield pyramidal signs, gait impairment, and distal dexterity loss; brainstem/cerebellar involvement causes ataxia, dysmetria, and oculomotor instability; sensory pathway damage impairs proprioception and coordination (140, 141). Phenotype and disease phase also matter: relapsing–remitting MS produces stepwise changes with partial recovery, while secondary or primary progressive MS accumulates fixed disability; heat sensitivity (Uhthoff's phenomenon), fatigue, and diurnal variability further modulate performance (142). Cognitive and visual comorbidities can compound motor control (143).

Rehabilitation standard of care is multidisciplinary and fatigue-first: programs combine task-specific PT, OT and SLT with energy-conservation and pacing strategies, temperature management (avoiding heat and using active cooling), and interval dosing at a moderate rating of perceived exertion (RPE 11–13 on the Borg 6–20 scale) to prevent post-exertional worsening (144–146). Gait/balance work emphasizes sensory reweighting and ataxia strategies (task decomposition, Frenkel-style coordination, external focus), with AFO or peroneal-nerve FES prescribed for foot drop and frequently re-titrated as fatigue fluctuates; dalfampridine may be considered to augment walking speed in eligible patient (147, 148). Upper-limb training prioritizes coordination and tremor mitigation (weighting, proximal stabilization, task-chaining) rather than pure strength alone; focal spasticity is managed with BoNT-A plus therapy/splinting within goal-based plans (149, 150). OT delivers ADL/IADL practice with graded difficulty and cognitive-compensatory strategies; bladder/bowel/sexual health and vision are addressed within the same pathway (151, 152). In MS, rehabilitation is typically organised in episodic, cyclic blocks triggered by relapses or functional decline. Supervised 4–6-week blocks (3–5 sessions per week, 45–60 min per session) combine task-specific PT, OT and SLT with aerobic training (≥30 min, 2 times per week) and resistance training (2 times per week), while home-based and telerehabilitation programmes are used to maintain dose between blocks (153). Intensity is fatigue-thresholded (Borg RPE 11–13) with interval dosing (5–10 min work, 2–3 min rest) and cool environments to limit heat sensitivity. Energy-conservation/pacing guides ADL/IADL work, and orthoses/FES are re-titrated as fatigue varies. Re-evaluation every 6–8 weeks (10MWT, 6MWT, TUG, 9HPT, MFIS) informs restarting new blocks after relapse or ≥10%–20% decline, while home and telerehabilitation programs maintain dose between cycles (144, 154). Ataxic or tremor-predominant phenotypes emphasize coordination and external-focus training (e.g., Frenkel drills, proximal stabilization), and foot-drop is managed with AFO or peroneal-nerve FES (147). General prognosis varies by phenotype and lesion load: patients with relapsing–remitting MS may recover for approximately 12 months after a relapse, whereas progressive forms show slow, relapse-independent decline. In contemporary MS cohorts, disability progression is commonly quantified using the Expanded Disability Status Scale (EDSS), a 0–10 ordinal scale in which a score of 3.0 indicates some functional limitation with independent ambulation and a score of 6.0 indicates the need for a unilateral walking aid to walk about 100 m. On this scale, median time from onset to EDSS 3.0 is approximately 10.7 years, and about 25% of patients reach EDSS 6.0 by 16 years; early cerebellar, brainstem or spinal-cord involvement and older age at onset are associated with poorer outcomes (147, 155). Guideline-based multidisciplinary rehabilitation and structured exercise improve function and health-related quality of life, with sustained benefits when integrated into home and tele-based follow-up (156).

#### 4.2.2 Role of neuroplasticity in the rehabilitation process

In MS, functional gains from rehabilitation come largely from activity-dependent neuroplasticity—the brain's capacity to reweight and re-route spared networks despite demyelination. Task-specific, repetitive, feedback-rich training has been shown to reshape functional connectivity on fMRI (e.g., strengthened motor and cerebellar-cortical networks) in parallel with clinical improvements after multidisciplinary and exercise programs; these changes are consistently interpreted as training-induced plasticity. Plasticity appears greater in earlier/less-severe disease and can be constrained by lesion burden, which helps explain heterogeneity of response. Exercise and structured rehab can also modulate biological mediators (e.g., ↑BDNF), supporting cognition and motor performance. Together, this is the biological substrate that rehab—and BCI-augmented protocols—seek to harness by precisely pairing motor intent with contingent sensory/assistive feedback to consolidate adaptive circuits (Hebbian timing). This preserved capacity for adaptive reorganization provides a neurophysiological rationale for employing BCI-based interventions to enhance or restore motor function in MS patient (157, 158).

#### 4.2.3 BCI use in MS

As described in Section 3.2, closed-loop BCIs that time-lock detected motor intention to immediate sensory or proprioceptive feedback provide the mechanistic basis for BCI-assisted rehabilitation. Because MS features fatigue and fluctuating conduction, BCI can still train/control when EMG is weak, using EEG motor-imagery or hybrid paradigms (e.g., P300/SSVEP) as a fatigue-resistant channel (159, 160). Aligned to this heterogeneity, we position brain–computer interface (BCI) by phenotype: when voluntary control permits, intention-contingent motor-imagery (MI) BCI is paired with functional electrical stimulation (FES)/robotics as a rehabilitative modality; if MI degrades with fatigue or conduction fluctuation, control switches to hybrid visual channels (P300/SSVEP) that remain usable despite weak electromyography (161). A representative gait program in MS delivered 24 sessions over eight weeks with MI-BCI-FES, improving Timed 25-Foot Walk speed and walking ability and showing earlier ERD onset; more generally, short intention-locked bouts with rests are used to accommodate MS-specific fatigability (161). Between supervised blocks, home/tele-BCI sustains training dose and participation, consistent with telerehabilitation benefits in MS (162). Effectiveness is evaluated on mobility/dexterity (Timed 25-Foot Walk, 10-Meter/6-Minute Walk, Nine-Hole Peg Test) and health-related quality of life (HRQoL) (MSQOL-54, MSIS-29); these measures are qualified/recommended for MS trials, and MI-BCI studies also report neural biomarkers (e.g., ERD/ERS) to guide assistance tapering (163). In MS, intention-contingent MI-BCI paired with FES for gait increased gait speed and walking ability, with concomitant improvements in quality of life (QoL). Moreover, because BCI modules can be deployed between supervised blocks via home- or tele-rehabilitation, intention-driven practice helps carry mobility gains into everyday participation, supporting QoL.

#### 4.2.4 BCI-based rehabilitation in MS: evidence and outcomes

In MS, BCI research has progressed from signal-level feasibility to demonstrations of how decoded brain activity can support clinically meaningful rehabilitation. Shiels et al. (164) examined EEG classification during executed and imagined hand movements in one person with MS and neurotypical controls and found that the MS participant achieved motor-imagery decoding accuracy comparable to healthy participants. Clinically, this shows that demyelination does not abolish usable motor-imagery patterns, meaning that MS patients can still provide a reliable “control signal” for MI-based BCIs. More recently, Russo et al. (165) extended this line of work to a larger group of people with MS and again reported motor-intention decoding accuracies in the same range as those of healthy controls. From a rehabilitation perspective, these studies together indicate that cortical motor networks in MS can still generate stable, decodable intention signals that are suitable for MI-BCI control, providing a neurophysiological basis for using MI-based BCIs in MS rehabilitation. Carrere et al. (161) implemented an eight-week gait program in nine people with relapsing–remitting or progressive MS (seven completers), using a motor-imagery–driven BCI to trigger FES during 24 walking sessions. Participants showed statistically and clinically meaningful gains in gait speed, walking ability and quality of life, and these behavioral improvements were accompanied by earlier onset and larger amplitude of motor-imagery–related event-related desynchronization (ERD), together with higher true-positive detection rates. Taken together, these neural changes indicate that repeated pairing of motor intention with FES-assisted gait made the motor-imagery signal stronger and more consistently decodable over time, which provides a plausible mechanistic link between BCI training and the observed functional gains. Complementary work by Tacchino et al. (166) showed that motor imagery in MS primes subsequent movement by increasing cortical excitability and sensorimotor activation, reinforcing the idea that MI-based BCIs are not only communication interfaces but also neuromodulatory tools that repeatedly engage and strengthen spared motor networks in MS.

#### 4.2.5 Limitations and future directions

However, existing BCI studies in MS are still at an early stage with respect to long-term efficacy. Most MI-BCI or BCI–FES trials use short intervention windows of about 4–8 weeks and report outcomes only at end of treatment or within a few weeks thereafter, so they primarily inform short-term effects. In the context of rehabilitation, we consider “long-term” to mean the durability of clinical and quality-of-life gains at least 6 months after the end of the intervention, with 12-month follow-up representing an ideal benchmark. Very few MS BCI studies have reported outcomes at this time scale, so the true long-term impact of these interventions remains uncertain.

In addition, the effectiveness of BCI appears to vary markedly across individuals because of heterogeneity in symptoms, lesion burden and disease duration, yet most published studies have been underpowered to explore moderators or stratified responder profiles. Quality-of-life (QoL) assessment has also been limited. Although some trials include basic QoL measures, comprehensive evaluations that capture key psychosocial dimensions such as fatigue, mood, motivation and social participation are often lacking. In MS, QoL responds most strongly to changes in fatigue, mood and participation, and exercise and telerehabilitation have been shown to improve QoL, whereas BCI-specific QoL evidence remains sparse. Future MS BCI trials should therefore pre-specify the Multiple Sclerosis Quality of Life-54 (MSQOL-54) and the Multiple Sclerosis Impact Scale-29 (MSIS-29) as core outcomes, include validated fatigue and mood scales, and assess the durability of effects at least 6–12 months after intervention completion (167). Larger, adequately controlled studies with longer follow-up are needed to confirm efficacy, to clarify which patients benefit most and to determine how BCI protocols can be integrated into routine MS rehabilitation.

### 4.3 Amyotrophic lateral sclerosis (ALS)

#### 4.3.1 Disease presentation and standard of care

Amyotrophic lateral sclerosis (ALS) is a fatal neurodegenerative disease of the central nervous system characterised by combined upper motor neuron (UMN) and lower motor neuron (LMN) dysfunction involving the brainstem and cervical, thoracic and lumbar spinal segments (168). Clinically, ALS causes progressive weakness of skeletal muscles involved in limb movement, swallowing (dysphagia), speech (dysarthria) and respiration, with patterns that vary according to the site of onset: limb-onset ALS typically begins with asymmetric distal weakness and atrophy, bulbar-onset disease presents with dysarthria and dysphagia, and respiratory-onset ALS causes early dyspnoea and reduced exercise tolerance (169, 170). Cognitive and behavioural changes occur early in approximately 35%–50% of patients, reflecting overlap with the ALS–frontotemporal dementia spectrum (171). As the disease progresses, many individuals eventually lose reliable motor communication and may enter a locked-in or complete locked-in state in which they are fully conscious but unable to communicate with the outside world (172).

Standard multidisciplinary management centers on symptom control, mobility preservation, and caregiver training. Core elements include task-specific PT/OT for transfer and ADL maintenance, speech and swallow therapy (SLT), respiratory physiotherapy, and energy-conservation/pacing strategies (173). Orthoses, lightweight wheelchairs, and non-invasive ventilation (NIV) are introduced as weakness progresses. Nutritional and psychosocial support are integral, as are regular multidisciplinary reviews to anticipate equipment needs (174). Rehabilitation is continuous and adaptive, rather than time-limited, with intensity scaled to disease stage: brief inpatient blocks or outpatient visits every few months, interspersed with home-based programs and tele-follow-ups. Median survival is 3–5 years from symptom onset (longer in limb-onset, shorter in bulbar-onset). Cognitive impairment (∼30%–50%) and respiratory involvement worsen outcomes. Disease-modifying drugs (riluzole, edaravone) modestly slow progression, but function eventually declines, making assistive and palliative integration essential (173, 175).

#### 4.3.2 Role of neuroplasticity in the rehabilitation process

Neuroplasticity does not halt motoneuron degeneration but supports compensation and short-term maintenance via collateral axonal sprouting and motor-unit remodeling, which temporarily enlarge surviving motor units and sustain force despite denervation (176). Centrally, patients show cortical reorganization with increased recruitment of premotor/supplementary motor areas and altered motor maps, consistent with a network-level shift that helps substitute for corticospinal dysfunction (177). Appropriately dosed, task-specific exercise can shape these plastic changes toward function, with randomized and meta-analytic evidence of benefits on mobility/ALSFRS-R compared with flexibility/usual care (178). At the same time, dosing must respect fatigability and respiratory reserve and avoid maladaptive plasticity (e.g., spasticity/co-contraction) that degrades movement efficiency (179).

#### 4.3.3 BCI use in ALS

In ALS, progressive degeneration of upper and lower motor neurons leads to paralysis while cognitive and sensory functions are often preserved, creating a “disconnected but aware” state (180). BCIs directly leverage this preserved cortical activity, enabling voluntary control and communication even when all peripheral motor output is lost. Compared with conventional PT/OT/EMG devices that depend on residual movement, BCIs use cortical activity itself as the control signal (21, 181). For early and mid-stage ALS, motor-imagery BCIs coupled with FES or VR systems can reinforce spared corticospinal circuits and help prolong voluntary control. In later stages, when limb and speech output are largely lost but gaze and sensory channels are still preserved, P300- or SSVEP-based BCIs can provide a practical communication and environmental-control option for many patients. However, as discussed below, once individuals progress to complete locked-in state (CLIS), even these non-invasive paradigms often fail to support stable communication (182, 183). By bypassing the failing motor neurons, BCIs shift ALS management from purely compensatory assistance to direct neural interaction, allowing preserved cortical activity to drive communication and environmental control (184, 185). Case reports and small cohort studies suggest that communication BCIs can stabilize or modestly improve patient-reported satisfaction with communication, perceived autonomy, and aspects of social participation over several months, even as motor function continues to decline (186). Thus, in ALS, BCIs appear to enhance specific domains of quality of life, most notably communication autonomy and the ability to maintain interaction between patients and caregivers, rather than reversing or halting the overall trajectory of functional decline; larger and longer-term studies are still needed to quantify the magnitude and durability of these effects.

BCI is phenotype-aligned: when voluntary or somatosensory resources are preserved, intention-contingent EEG paradigms can be used for training/control; where EMG is weak or endurance is limited, P300/SSVEP or hybrid channels provide robust, low-effort control that remains usable despite fluctuating conduction (187). In later or high-fatigue stages, BCI is positioned primarily as an assistive interface for communication and environmental control, including sustained home use and, in selected cases, fully implanted systems for locked-in ALS (188). Pragmatic dosing uses short, interval-based sessions (≈20–30 min with rests) to manage attentional load and fatigue typical of ALS/visual BCIs, with progression guided by online neural/decoder performance (e.g., accuracy, confidence) (189).

Within the standard multidisciplinary care pathway, brain-computer interfaces (BCIs) serve two ALS-specific roles. First, as an early communication backup, often introduced in parallel with eye-tracking while gaze remains usable, so that a reliable, home-deployable channel is ready before bulbar or oculomotor failure—with feasibility and durability demonstrated from P300 spellers to independent home use and a fully implanted at-home system (187). Second, as targeted practice in early limb-onset disease when kinesthetic imagery is dependable: motor-imagery (MI) BCI may cue task-oriented FES/robotics in short, fatigue-aware intervals (≈10–20 min bouts with rests, 3–5×/week), with progression gated by decoder stability and respiratory reserve, consistent with ALS rehabilitation principles and ongoing BCI-robotics development (190). As lower-motor-neuron denervation progresses—or imagery/gaze becomes unreliable—programs pivot to low-effort hybrid BCIs (P300/SSVEP) used primarily as assistive technology for communication and environmental control, typically home-based and caregiver-supported (191). Endpoints reflect the role: rehabilitative trials emphasize ALSFRS-R motor/bulbar subscores and kinematic/dexterity measures, whereas assistive deployment prioritizes communication rate/accuracy, daily device-on time, independence, and caregiver time saved (192).

#### 4.3.4 BCI-based rehabilitation in ALS: evidence and outcomes

In the last five years, ALS-focused BCI research has primarily aimed to restore or enhance communication by translating neural activity into text or synthesized speech. N. S. Card et al. (193) implanted an intracortical electrode array in the ventral precentral gyrus of a person with ALS and showed that attempted speech could be decoded with high accuracy over more than eight months of home use, allowing the participant to hold self-paced conversations at a functionally useful rate despite severe paralysis. In a complementary approach, Luo et al. (194) used a chronically implanted electrocorticography (ECoG) array over ventral sensorimotor cortex to decode six intuitive speech commands in a person with severe dysarthria due to ALS, allowing reliable control of computer applications over a three-month period without recalibration. Together, these studies illustrate that both intracortical and ECoG-based BCIs can transform preserved cortical speech-related activity into practical communication channels, thereby supporting autonomy and participation in daily life for people with ALS. Beyond short-term feasibility studies, a few reports have examined the stability and home use of BCIs in ALS. Vansteensel et al. (195) described a person with late-stage ALS who used an implanted BCI independently at home for around seven years without major technical failures, showing that continuous, self-directed use is feasible under real-life conditions. Complementary work by Wyse-Sookoo et al. (196) demonstrated that speech-related electrocorticographic (ECoG) signals from a chronically implanted array remained stable over at least 12 months. From a rehabilitation and assistive-technology perspective, these findings suggest that invasive ECoG-based systems can provide stable neural signals to support communication BCIs over periods of about 12 months up to approximately seven years in people living with severe paralysis.

Although existing studies confirm the potential of BCI technology to restore or improve communication in many people with ALS, results in complete locked-in state (CLIS) are far less encouraging. Chaudhary et al. (197) reported that, despite repeated attempts with electroencephalography (EEG) and functional near-infrared spectroscopy (fNIRS) based BCIs, several patients with CLIS were unable to establish reliable communication. Similarly, Pires et al. (198) tested a visuo-auditory P300 paradigm that achieved high online accuracy in healthy participants but performed only at near-chance levels in fully locked-in ALS. From a rehabilitation standpoint, these findings suggest that current non-invasive BCI systems, which rely on sustained attention, intact sensory pathways and some residual capacity to engage with external stimuli, may be insufficient once patients reach complete lock-in. Researchers have also begun to combine BCIs with augmented reality and eye-tracking to make communication systems more portable and acceptable in everyday life for people with ALS. Li et al. (199) developed a wearable P300 speller implemented in a mixed-reality headset and showed that, compared with a conventional screen-based BCI, the mixed-reality system elicited larger P300 responses and achieved higher spelling accuracy and information transfer rates. From a rehabilitation perspective, this indicates that a compact, head-mounted BCI can maintain good decoding performance while improving portability and user acceptance, making it more suitable for home-based communication support in ALS.

Several preliminary studies have provided early examples of adaptive and patient-centred BCI designs that are relevant for ALS. For example, Aliakbaryhosseinabadi et al. (200) developed an online EEG-based BCI that continuously estimates the user's attentional state and automatically adjusts channel/classifier selection and feedback when vigilance fluctuates. Such attention-aware adaptation could reduce cognitive effort and help maintain stable BCI performance during prolonged or fatiguing sessions, which is particularly important for people with ALS. Zou et al. (201) used deep-learning methods to decode eye-related biosignals into words and implemented their algorithms on a portable platform, illustrating how modern machine-learning and low-power hardware can support communication interfaces that are accurate yet lightweight enough for home and community use. This line of work points toward future hybrid systems that combine robust decoding with portable, user-friendly devices to better support communication needs in people with severe motor paralysis, including those with ALS. Beyond technical development, patient perspectives also highlight the promise of BCI applications. A large-scale questionnaire survey supported by the Japan ALS Association revealed that patients with ALS expressed strong expectations for BCI technologies to help overcome their “motivational difficulties”—that is, the inability to translate intention into voluntary movement due to severe motor paralysis, reflecting the practical clinical needs and the broad acceptance of this emerging assistive approach among patients (202, 203).

#### 4.3.5 Limitations and future directions

As ALS progresses, neural function deteriorates and most BCI evidence remains confined to small case series or single-participant reports. Although long-term feasibility of BCI use has been demonstrated in isolated cases (e.g,202-203), larger and more diverse cohorts are needed to systematically evaluate the stability, durability and scalability of ALS BCIs over months to years. Individual differences in the pattern and rate of neurodegeneration mean that BCI systems need to adapt to each patient's evolving brain and clinical status, yet current research has not adequately addressed personalisation or robust adaptivity. A few exploratory studies have begun to develop adaptive BCI approaches that monitor cognitive state or signal quality and adjust decoding strategies or feedback in real time, but these remain early prototypes tested in small samples and over short time frames, and there is still no clinically validated adaptive framework that works reliably across the full spectrum of ALS severity. Future work should therefore focus on systematically designing and validating adaptive ALS BCIs that can operate under the extreme neurophysiological constraints of advanced disease and remain usable across stages of progression, while also integrating seamlessly into multidisciplinary care.

The application of BCI technology often requires patients to undergo extensive, repetitive training to achieve reliable control, as users must sustain focused attention, perform consistent motor imagery and learn to modulate neural activity in response to feedback. These cognitive and mental demands can induce fatigue even in healthy participants, and are accentuated in advanced ALS, where progressive motor neuron degeneration, respiratory impairment and pervasive fatigue make it difficult to maintain concentration or perform repeated mental tasks for long periods. In addition, reduced voluntary muscle control, including oculomotor limitations, can hinder effective interaction with visual or auditory feedback during training. These disease-specific factors increase the cognitive load associated with BCI use and highlight the need for shorter, fatigue-sensitive, individually tailored training protocols and hybrid paradigms that can flexibly switch between control channels as capacity changes. Finally, there is a relative lack of research on how BCIs affect psychological and emotional state, caregiver burden and broader social functioning in ALS. Future ALS BCI trials should incorporate standardised measures of health-related quality of life, mood, motivation, social participation and caregiver outcomes, so that the development of advanced BCI technology is aligned with the comprehensive health needs of people living with ALS.

### 4.4 Spinal cord injury (SCI)

#### 4.4.1 Disease presentation and standard of care

Spinal cord injury is an injury to the spinal cord caused by trauma (e.g., falls and road traffic injuries) or non-traumatic causes (e.g., tumors, degenerative and vascular diseases, infections, toxins, or birth defects) (204). The disorder can result in complete or incomplete sensory or motor loss below the plane of injury, with the degree of associated impairment depending on the severity of the injury and the location of the injury (205). The disorder can severely limit the ability to perform daily activities, making it difficult for patients to achieve independent social participation. Spinal cord injury is a leading cause of long-term disability, with an estimated 250,000 to 500,000 new injuries each year (206, 207). Spinal cord injuries interrupt communication between the brain and the spinal cord region that produces movement, leading to paralysis. BCI technology offers new motor reconstruction options for people with spinal cord injuries by establishing a digital bridge between the brain and spinal cord to restore communication, decoding neural activity captured via EEG, ECoG, sEEG, or microelectrode arrays (MEAs) into commands for external device control.

Standard SCI rehabilitation is multidisciplinary and begins as soon as patients are medically stable. Acute inpatient rehabilitation focuses on prevention of complications (pressure injury, contracture, respiratory infection, thrombosis) and early mobilization. The subacute and chronic phases emphasize task-specific PT and OT for bed mobility, transfers, wheelchair skills, and ADL retraining. Gait training (e.g., body-weight-supported treadmill or robotic exoskeleton), FES cycling, strength and endurance exercise, and upper limb robotics are key components to promote neuroplasticity and functional restoration (208). Speech, bladder, bowel, and sexual function programs address secondary impairments. Standard care includes psychological support, spasticity management (BoNT-A or oral antispastics), orthotic prescription, and community reintegration (209). Inpatient rehabilitation typically lasts 8–12 weeks for incomplete injuries and longer for complete lesions, followed by outpatient or home programs extending several months. Training frequency is usually 5 days per week, ≥3 h/day of mixed physical and functional practice, consistent with intensity guidelines for neurorehabilitation (210, 211). Telerehabilitation and home exercise programs are increasingly used to maintain dose continuity between supervised phases, with re-evaluation every 6–8 weeks (e.g., SCIM, WISCI-II, 10MWT). Recovery potential depends on neurological level, completeness, and time since injury. Most recovery occurs within 6–12 months, though incomplete SCI can show delayed improvements up to two years (212). Better outcomes correlate with AIS C–D grades, younger age, and early intensive rehabilitation. Complete injuries (AIS A) rarely regain ambulation but benefit from compensatory independence training and assistive devices (213).

#### 4.4.2 Role of neuroplasticity in the rehabilitation process

After spinal cord injury (SCI), recovery is mediated by activity-dependent neuroplasticity in spared cortico- and propriospinal circuits; rehabilitative training shapes this spontaneous reorganization toward functional networks rather than “repairing” severed tracts (214). Task-specific, high-repetition stepping/walking practice produces larger locomotor gains than impairment-focused exercise, aligning with clinical guidelines that prioritize task-specific walking training for motor-incomplete SCI (215). Plasticity can be targeted to spinal pathways: operant conditioning of the H-reflex remodels reflex circuits and improves locomotion in animals and people with incomplete SCI (216). Spike-timing–dependent approaches such as paired corticospinal–motoneuronal stimulation (PCMS)/paired associative stimulation enhance corticospinal transmission and upper-limb motor outcomes in chronic and subacute SCI (217). Neuromodulation can create a permissive state that amplifies training: targeted epidural spinal stimulation restored volitional walking in chronic SCI, and a brain–spine interface (digital bridge) enabled natural overground walking in community settings (218). Spared corticospinal and propriospinal circuits undergo activity-dependent reorganization, forming new synaptic relays around the lesion. Repetitive, task-specific training and neuromodulation (e.g., FES, tDCS, or BCI-driven stimulation) can enhance cortical excitability, promote synaptic strengthening, and facilitate reconnection to spinal interneurons (219). Thus, neuroplasticity underlies both spontaneous and training-induced recovery. BCIs exploit preserved cortical intent signals to re-establish sensorimotor loops.

#### 4.4.3 BCI use in SCI

In spinal cord injury, these mechanisms are applied in the context of preserved cortical motor areas and disrupted descending pathways. Because people with spinal cord injury (SCI) commonly exhibit fatigability, fluctuating spasticity, and—when the lesion is at or above T6—risk of autonomic dysreflexia and orthostatic hypotension, BCI sessions are delivered as short intention-locked bouts (≈20–30 min) with brief rests, 3–5 times per week fits routine rehabilitation intensity and permits adjunctive layering onto standard therapy, augmenting intention-locked practice without replacing existing care (220). Home- or tele-BCI maintains training dose between supervised blocks, preventing skill regression (221). In these SCI protocols, neurophysiological biomarkers derived from sensorimotor EEG and corticospinal excitability (e.g., changes in motor-imagery–related desynchronization or TMS-evoked motor potentials) are used to titrate FES or exoskeleton assistance and task difficulty, gradually shifting from more passive guidance to increasingly active, intention-driven practice as residual pathways strengthen (222, 223).

Brain–computer interface (BCI) use has improved quality of life (QoL) through three converging routes: (1) restoring functional movement—intention-contingent BCI–FES and brain–spine interfaces have enabled previously impossible actions (e.g., self-feeding, reaching–grasping, and community ambulation), with studies reporting meaningful QoL gains alongside new independence in activities of daily living; representative demonstrations include intracortical BCI-FES for reach–grasp in chronic tetraplegia and a digital brain–spine bridge that translated to day-to-day walking and better perceived QoL (224). (2) alleviating central neuropathic pain, a major QoL determinant in SCI—home-deployed, patient-managed EEG-BCI neurofeedback has reduced pain intensity and improved related QoL domains in clinical studies (221). (3) supporting participation at home, where month-to-months BCI use is feasible and users/caregivers rate benefit as outweighing burden, citing enhanced interaction and social engagement—effects that map directly onto QoL (225).

Sensorimotor involvement varies by neurological level (cervical vs thoracic/lumbosacral), completeness (AIS A–D), and incomplete syndromes (e.g., central cord, Brown-Séquard, anterior/posterior cord), which differentially affect hand/arm control, trunk stability, gait, and sensory/autonomic function (226). Aligned to this heterogeneity, brain–computer interface (BCI) is positioned for cervical AIS C–D to drive task-relevant hand function by pairing intention-contingent decoding with FES/robotics (restoring reach–grasp in tetraplegia), while brain–spine interfaces and spinal neuromodulation support stepping when supraspinal drive is limited (224). Pragmatic dosing follows established SCI rehabilitation patterns: locomotor training commonly 3–5 sessions/week for ∼25–50 min/session over ∼8 weeks; for upper limb, the multicenter ARC-EX trial delivered ∼25 sessions over 2 months and improved strength, dexterity and sensation versus rehabilitation alone (227).

#### 4.4.4 BCI-based rehabilitation in SCI: evidence and outcomes

In the field of spinal cord injury (SCI), BCI systems have been explored both as assistive devices—to compensate for lost motor function by externally controlling effectors—and as rehabilitative tools aimed at promoting neuroplastic recovery within spared neural pathways. For example, Jorge et al. (228) implanted microelectrode arrays into the hand area of motor cortex in a person with chronic C5/6 AIS B tetraplegia and showed that attempted single-finger movements could be decoded with high accuracy. Although no external device was controlled in that study, it demonstrates that well-defined, finger-specific motor commands remain accessible years after cervical injury and could, in principle, be used to drive grasp-assist devices or computer interfaces. In a related proof-of-concept study, Pulferer et al. (229) showed that continuous two-dimensional movement trajectories could be decoded from attempted arm movements in a person with SCI, providing a basis for continuous control of cursors, wheelchairs or robotic effectors. Together, these experiments highlight that preserved cortical motor signals in SCI are rich enough to support both discrete and continuous assistive control, which is a prerequisite for future BCI-based rehabilitation and mobility systems. In contrast, the Brain–Spine Interface (BSI) developed by H. Lorach et al. (230) exemplifies a hybrid neuroprosthetic approach, it succeeded in restoring communication with a digital bridge between the brain and spinal cord that enabled an individual with chronic tetraplegia to stand and walk naturally in community settings, and even cope with complex terrain, re-establishing the patient's motor control function. the study demonstrates that, years after spinal cord injury, preserved cortical motor commands can still be harnessed to re-engage spinal locomotor circuits, enabling task-specific gait training and functional ambulation. On the rehabilitation side, Colamarino et al. (231) tested a BCI-supported motor imagery (MI) programme in patients with traumatic cervical SCI in the subacute phase and reported improvements in hand sensorimotor function, interpreting these gains as evidence that MI-BCI can engage early brain and spinal plasticity to support upper limb recovery. Jovanovic et al. (232) likewise evaluated a BCI-triggered functional electrical stimulation therapy (BCI-FEST) for reaching and grasping in individuals with subacute SCI and observed clinically relevant improvements in arm function, supporting BCI–FES as a promising adjunct to standard upper-limb rehabilitation rather than a replacement. Collectively, these pilot studies suggest that BCI can either substitute lost motor pathways as an assistive interface or promote neuroplastic recovery as a rehabilitative tool. While most remain preliminary, emerging clinical evidence points toward increasing feasibility for both approaches in spinal cord injury.

Meanwhile, BCI technology based on SCI rehabilitation is also improving. For example, Davis et al. (233) developed a portable, modular BCI platform that can be mounted on a wheelchair and used to control an assistive hand device in the home environment, illustrating how rehabilitation-oriented BCIs can be engineered for real-world use and integrated into daily activities rather than remaining confined to the laboratory. At the algorithmic level, Xu et al. (234) showed that a graph-convolution–based deep-learning framework can improve EEG motor-imagery decoding performance, which is directly relevant for SCI rehabilitation because more accurate and robust intention decoding is a prerequisite for reliable BCI control of FES, exoskeletons and other assistive devices.

In addition, there are also studies evaluating the acceptance, feedback on the use, and clinical feasibility of BCI technology in spinal cord injury patients, for example, L. Ferrero et al. (235) evaluated the acceptance and user experience of MI-based BMI and lower extremity exoskeletons the use of in patients with incomplete SCI, and the study demonstrated that the patients experienced satisfaction with the use of the exoskeletons, and that the level of mental and physical loading was within reasonable limits. X. Chen et al. (236) conducted a clinical trial in which ten individuals with severe spinal cord injury used a non-invasive, P300-based BCI to drive a powered wheelchair, demonstrating that reliable, intention-driven wheelchair control is feasible in this population. This work suggests that BCI-controlled wheelchairs could extend rehabilitation outcomes beyond impairment-level gains toward greater real-world mobility and autonomy.

#### 4.4.5 Limitations and future directions

Currently, the trend of research in this field is to combine advanced machine learning algorithms and deep learning models to improve the accuracy and real-time performance of signal processing. However, there are still some gaps in the existing research, including the impact of individual differences, the lack of long-term adaptability, and the lack of deeper understanding of physiological feedback mechanisms. These challenges suggest that more in-depth exploration is urgently needed in the application of BCI technology in practice. The adaptability and stability of existing BCI systems in long-term use have not yet been fully verified. In this context, long-term refers not only to performance stability over extended continuous use—potentially spanning multiple years—but also to the biological durability of implanted electrodes, signal consistency, and sustained user engagement. Although a few exceptional cases have demonstrated reliable intracortical BCI operation for up to a decade (237), systematic evidence across larger populations and diverse devices remains limited. Therefore, improving the long-term adaptability of both hardware and software, while maintaining user motivation and participation, remains an urgent challenge for clinical translation. In addition, insufficient understanding of the physiological feedback mechanisms underlying neuroplastic recovery limits the optimization of BCI-based rehabilitation. In particular, it remains unclear how different forms of feedback—such as visual, proprioceptive, tactile, or functional electrical stimulation—modulate cortical excitability and sensorimotor reorganization. A deeper understanding of these closed-loop interactions between neural activity and peripheral feedback is essential for designing more effective and adaptive BCI systems for motor rehabilitation. Future research should focus on the development of personalized BCI systems and deeply explore the influence of physiological feedback mechanisms on exercise rehabilitation. In addition, optimizing the user experience and improving the real-time and stability of the system are also important research directions.

### 4.5 Parkinson's disease (PD)

#### 4.5.1 Disease presentation and standard of care

Parkinson's disease (PD) is a common neurodegenerative disorder characterized by the loss of dopamine neurotransmitters in the brain, particularly the degeneration of dopaminergic neurons in the substantia nigra (238), leading to symptoms of motor dysfunctions such as tremor, rigidity, bradykinesia, and postural instability (239). Over the last 30 years, an increasing trend in the age-standardized rate (ASR) of PD has been observed in most cases (240) and its prevalence has steadily increased with age (241).

In this review, we use “brain–computer interface (BCI)” to denote systems that decode brain activity to deliver external feedback or assistive/rehabilitative control (e.g., EEG motor-imagery BCIs and neurofeedback). By contrast, deep-brain stimulation (DBS)—including adaptive/closed-loop DBS (aDBS) that uses beta-rhythm sensing to adjust stimulation in real time—is treated as neural-signal–guided neuromodulation rather than BCI in PD clinical parlance.

The current standard of care primarily involves pharmacological therapy (e.g., levodopa and dopamine agonists), physical and occupational therapy to maintain motor function, and in advanced cases, deep brain stimulation (DBS) of the subthalamic nucleus or globus pallidus (20). DBS is a widely accepted neuromodulatory treatment that can alleviate motor symptoms by regulating abnormal *β*-band oscillations. Recent studies have further advanced toward adaptive closed-loop DBS, which automatically adjusts stimulation intensity based on real-time monitoring of *β*-rhythm fluctuations to optimize motor outcomes and minimize side effect (20, 242, 243). Building on the definitions above, PD care increasingly adopts brain-signal–guided methods along two complementary tracks: (i) adaptive/closed-loop DBS that titrates stimulation from real-time beta-band signals to refine neuromodulation (244, 245); and (ii) BCI modules that decode brain activity to deliver intention-contingent feedback or assistive control during rehabilitation. We thus keep closed-loop DBS under neuromodulation and BCI under rehabilitative/assistive control to avoid terminological ambiguity. Rehab is episodic and ongoing across the disease course, but individual blocks commonly run 2–12 weeks at 2–3 sessions/week in clinical trials. Protocolized programs include LSVT BIG/LOUD: 4 sessions/week for 4 weeks (16 visits) with daily home practice; many centers use 6–12-week outpatient blocks for gait/balance with maintenance via home/telerehabilitation. Inpatient bursts of 2–8 weeks (or phased models such as ∼2 months inpatient followed by telerehabilitation) are used for complex cases, post-hospitalization, or peri-DBS optimization (246). Parkinson's disease is a slowly progressive disorder with heterogeneous trajectories: most patients live for many years, but overall survival is modestly reduced versus the general population (pooled standardized mortality ratio ≈ 1.6) (247). Prognosis depends strongly on age at onset, phenotype, and cognition—people presenting with normal cognition can approach near-normal life expectancy, whereas early cognitive impairment and Parkinson's disease dementia (PDD) are associated with shorter survival (248). Longitudinal cohorts show that the risk of dementia rises with disease duration (median ≈ 15 years from diagnosis; cumulative prevalence ≈ 83% at 20 years), making cognitive decline a major driver of later disability and institutionalization (249). Clinical milestones such as hallucinations, recurrent falls, and dementia typically accumulate over time and are tightly linked to reduced independence and survival (250). Phenotypic features (e.g., postural-instability/gait-difficulty presentation) further signal higher risks of cognitive decline and complications (251).

#### 4.5.2 Role of neuroplasticity in the rehabilitation process

In Parkinson's disease (PD), clinically meaningful improvement hinges on activity-dependent neuroplasticity within cortico–basal-ganglia–thalamo–cortical loops. Dopamine normally gates corticostriatal LTP/LTD, and dopamine loss in PD disrupts physiological synaptic plasticity, undermining motor learning capacity (252). Human TMS/PAS studies show that LTP-like motor-cortex plasticity is reduced off medication and can be restored by levodopa (in non-dyskinetic patients), linking dopaminergic state to the brain's capacity to relearn movements (253). Rehabilitation that is goal-based, task-specific, aerobic, and feedback-rich leverages this residual capacity: preclinical and human data indicate exercise enhances synaptic/neurotrophic signaling (e.g., BDNF), reorganizes motor networks, and supports motor skill acquisition, while randomized clinical trials show aerobic/high-intensity exercise is safe/feasible and attenuates motor signs (254). Neuromodulation further shapes plasticity: subthalamic deep-brain stimulation (STN-DBS) modulates motor-cortex plasticity, and pairing STN stimulation with TMS can induce cortical plasticity, suggesting synergistic windows for training (255). In sum, PD recovery strategies work by restoring and directing plasticity—optimizing dopaminergic tone, then dosing intention-driven practice (and, where indicated, DBS) to re-weight circuits that support faster, larger, and more automatic movement (256).

#### 4.5.3 BCI use in PD

Truly brain-signal–based approaches provide intention/biomarker-contingent therapy that conventional cueing or therapist-timed practice cannot. In a proof-of-concept randomized controlled study, adding real-time fMRI neurofeedback—providing feedback and coaching to up-regulate activity in motor-related cortex—to otherwise identical motor practice produced greater improvements in motor function than practice alone. In another study using implanted electrodes to record local field potentials from the subthalamic nucleus (STN), patients achieved “*β*-neurofeedback,” volitionally suppressing pathological *β* oscillations and exhibiting faster movement initiation. Taken together, these findings indicate that brain-contingent training can directly modulate disease-relevant abnormal neural activity and strengthen plasticity within the target circuits (257). When visible movement is small or unreliable, EEG motor-imagery BCIs can still be used in PD and paired with assistive feedback (e.g., FES or robotics), so that the timing and amount of assistance are controlled by the decoded motor-intention signal instead of relying only on residual visible movement (258). Widely used devices serve different triggers and goals: deep brain stimulation (DBS/aDBS) delivers implanted neuromodulation for symptom control (259), external cueing systems (auditory/visual/inertial sensors) time gait without reading brain signals (260), and assist-as-needed robots or treadmills scale practice from kinematics rather than neural state (261). BCIs can be layered on top of these approaches to drive circuit-level learning and more personalized progression when visible movement is small or inconsistent (259).

In PD, decoding-based BCIs influence quality of life (QoL) primarily through motor-function–mediated gains in mobility and participation: real-time neurofeedback enables patients to volitionally modulate target neural biomarkers—up-regulating motor-area activity with fMRI-NF or suppressing pathological *β* bursts cortically/subcortically, which translates to faster movement initiation and superior motor performance versus matched practice without feedback; improvements in motor severity and gait metrics are, in turn, linked to better PD-specific QoL (PDQ-39/PDQ-8) across mobility and activities-of-daily-living domains (257).

Parkinson's disease shows marked sensorimotor heterogeneity across motor phenotypes (tremor-dominant versus postural-instability/gait-difficulty, PIGD), further modulated by cognitive and visual comorbidities, medication ON/OFF fluctuations, and fatigue (262). In practice, these axes guide BCI selection: when motor imagery is preserved, patients can use intention-contingent MI-BCIs (for example, fMRI- or EEG-guided neurofeedback) to enhance activity in motor cortical regions during training, with randomized and pilot trials showing superior motor gains compared with matched practice without feedback (263). If motor-imagery–related rhythms become unstable in the context of PIGD, freezing of gait, or pronounced fatigability, visually evoked paradigms (P300/SSVEP) offer a more robust control channel, a positioning supported by PD-focused BCI reviews and evidence of visual pathway involvement in PD (264). When deep brain stimulation (DBS) leads are present, beta-targeted neurofeedback and adaptive/closed-loop DBS use real-time *β* sensing to accelerate movement initiation and individualize stimulation, providing a neuromodulatory complement to decoding-based BCIs (265).

#### 4.5.4 BCI-based rehabilitation in PD: evidence and outcomes

Within this clinical context, BCI technologies are emerging as complementary tools to enhance neuromodulation and rehabilitation. M. Abtahi et al. (266) combined electroencephalography (EEG) and functional near-infrared spectroscopy (fNIRS) with inertial-sensor motion capture and a sensorized glove to record cortical activity together with both gross limb movements and fine finger kinematics. This multimodal monitoring framework illustrates how BCI-oriented systems can link disease-relevant brain signals to quantitative movement behaviour, providing a basis for future rehabilitation protocols that adapt feedback or assistance according to both neural and kinematic state. M. Jochumsen et al. (267) analysed EEG and electromyography (EMG) during repeated wrist and ankle movements in nine people with Parkinson's disease and trained classifiers that distinguished movement-related activity from rest with accuracies of approximately 88%–89%. From a rehabilitation standpoint, these findings suggest that combined EEG–EMG biomarkers can reliably detect movement intention or tremor-related activity in Parkinson's disease, which is a prerequisite for real-time BCI systems that trigger cueing, functional electrical stimulation or robotic assistance in a functionally meaningful way. Similarly, Miladinovic et al. (258) delivered 14 sessions of lower-limb motor-imagery BCI training to seven patients with Parkinson's disease and showed that EEG-based decoders could distinguish imagined movements from rest with accuracies well above chance, meaning that participants were able to operate the MI-BCI reliably. This result implies that people with Parkinson's disease can generate usable intention signals for lower-limb BCIs, supporting the development of gait- and posture-oriented BCI interventions.

#### 4.5.5 Limitations and future directions

While traditional pharmacological and physical therapies remain the mainstay of PD management, their long-term effectiveness is limited. BCI systems therefore offer a novel pathway for closed-loop neurorehabilitation—by decoding cortical activity and translating neural signals into real-time control commands to drive assistive devices, robotic systems, or adaptive stimulation protocols. Future research should focus on integrating BCI decoding with adaptive DBS frameworks, enabling cortical signals and *β*-band oscillations to jointly guide personalized, closed-loop neuromodulation for more precise and responsive PD rehabilitation.

### 4.6 Cerebral palsy (CP)

#### 4.6.1 Disease presentation and standard of care

Cerebral palsy (CP), defined as a group of nonprogressive disorders of movement and posture, is the most common cause of severe neurodisability in children, and which are characterized by motor deficits with or without perceptual and intellectual deficits (268). CP is a highly heterogeneous condition, encompassing subtypes such as spastic, dyskinetic, and ataxic forms, each associated with distinct neural injury patterns and rehabilitation needs (269). This clinical variability requires individualized therapeutic strategies rather than uniform rehabilitation protocols.

Standard rehabilitation for children with CP is multidisciplinary, typically involving physical therapy, occupational therapy, and speech therapy, sometimes in combination with functional electrical stimulation (FES), robotic-assisted training, and constraint-induced movement therapy (CIMT) (270, 271). These interventions aim to reduce spasticity, improve coordination and gait, and enhance functional independence. Treatment is most effective when initiated during early childhood (approximately before age five), when neuroplasticity is at its peak (272). Typical programs involve 3–5 therapy sessions per week, each lasting 45–60 min, often continued over several months or years. Treatment selection depends on CP subtype (spastic, athetoid, ataxic), severity, and cognitive function, which collectively guide the timing, intensity, and modality of intervention (273). Cerebral palsy is lifelong and non-progressive, but outcomes are highly variable across children (274). Most children follow predictable gross-motor growth curves and reach a motor peak in early childhood (≈5–7 years) with subsequent stabilization at a level determined largely by GMFCS (gross motor function classification system) severity (275). By adolescence, independent ambulation is common in milder CP (GMFCS I–II), GMFCS III typically walk short distances with aids and use a wheelchair for community mobility, and GMFCS IV–V rely mainly on wheeled mobility, especially outdoors (276). Their overall health depends more on comorbidities (e.g., feeding/aspiration risk, epilepsy, respiratory complications) than on motor function level alone (277, 278). If early intervention is properly implemented, assistive devices are suitable, and educational and communication support is provided, many children can achieve good levels of participation and quality of life. However, parents and the team need to re-evaluate the goals and expectations at key age milestones (such as school entry, adolescence, and transition to adulthood), and promptly adjust the plan (279).

#### 4.6.2 Role of neuroplasticity in the rehabilitation process

Neuroplasticity refers to the brain's tendency to strengthen frequently used, correctly coordinated pathways and to down-weight underused or maladaptive pathways (280). In cerebral palsy (CP), injury occurs while neural circuits are still being established; thus, rehabilitation is not about “repairing the lesion” but about re-engaging and optimizing spared networks through training (281). Effective intervention follows four motor-learning principles—adequate dose (high repetition), task specificity, timely feedback, and appropriately titrated intensity/timing—which are operationalized in task-oriented programs for CP (282). In practice, meaningful functional goals are decomposed into smaller, attainable components, deliver training in a closed loop of intention, correct execution, and immediate feedback, repeated across many successful trials (283). This process gradually remaps cortical representations, rebalances interhemispheric excitation–inhibition, reduces abnormal co-contraction and mirror movements, and stabilizes intent–muscle coupling (281). Functionally, children achieve steadier sitting, more accurate grasp, independent standing, and more continuous gait (284). A range of technologies can “make the right pathway succeed”—for example constraint-induced or bimanual intensive therapy, robotics or functional electrical stimulation, and excitability-biasing adjuncts (e.g., pediatric rTMS/tDCS), with BCI-based neurofeedback emerging to provide brain-state–contingent feedback in select programs (285).

#### 4.6.3 BCI use in CP

However, traditional therapies often face challenges including limited long-term engagement, plateaued progress, and insufficient adaptation to individual variability in symptom profile, motivation, and cognitive ability. Unlike adults, children possess a broader window of neuroplasticity, enabling more extensive cortical reorganization and cross-hemispheric compensation following brain injury (286, 287). This developmental advantage provides a strong rationale for integrating brain–computer interface (BCI) systems into pediatric rehabilitation. Given children's heightened neuroplasticity, BCI systems can be used to link motor-related brain activity to task-relevant feedback (as outlined in Section 3), thereby supporting long-term engagement and motor learning in pediatric rehabilitation (288). Moreover, rehabilitation goals vary by CP subtype: Spastic CP emphasizes muscle tone regulation and selective control; Ataxic CP focuses on coordination and balance; Dyskinetic CP targets suppression of involuntary movement (289). BCI technology can provide adaptive treatment for children with different needs. Standard assistive and training devices—orthoses (e.g., AFOs), walkers/standers and gait trainers, robotic exoskeletons, and neuromuscular/functional electrical stimulation—primarily act on the body and biomechanics to provide alignment, safety, and high-repetition practice under therapist or device control rather than brain-state control (290). By contrast, BCI systems operate at the neural level and deliver feedback contingent on motor-related brain activity, with the specific aim in CP of promoting circuit re-weighting and more selective motor control rather than diffuse co-contraction (288). In practice, BCIs are used as adjuncts layered onto conventional programs—often pairing the decoded intent with FES/robotics/VR to time assistance to brain activity when overt movement is weak or poorly selective, while standard devices continue to supply the necessary mechanical support and dose. This complementarity aligns with contemporary CP care frameworks that emphasize task-specific, feedback-rich, and developmentally timed intervention, with technology chosen according to the child's motor goals and context (290).

Decoding-based BCIs can influence quality of life (QoL) through two primary routes: communication access and motor function training. For children with severe dysarthria or limited manual ability, P300/MI BCIs provide alternative access for communication and interaction, which pediatric BCI reviews and empirical work link to greater participation and perceived autonomy—key drivers of QoL (291). On the motor side, BCI-triggered functional electrical stimulation (BCI-FES) and related MI-BCI paradigms are feasible and safe in hemiparetic CP and have shown improvements in hand control accompanied by cortical neurophysiologic change, a mechanism that plausibly translates to independence in daily activities and caregiver burden reduction (292). Emerging home/gamified BCI modules aim to sustain training dose and engagement outside the clinic—thereby addressing access barriers that matter for family QoL—and are being formally evaluated in pediatric cohorts (291). To document QoL impact rigorously, CP-specific instruments such as CP QOL-Child/Teen and CPCHILD are validated and recommended endpoints for trials and programs that embed BCI in rehabilitation pathways (293).

Sensorimotor involvement varies by lesion site/extent and developmental timing: periventricular white-matter injury typically drives spastic phenotypes, basal-ganglia/thalamic injury underlies dyskinetic CP, and cerebellar involvement contributes to ataxic presentations; superimposed corticospinal reorganization (including ipsilateral projections from the non-lesioned hemisphere) reflects injury during a high-plasticity window and helps explain selective motor control deficits (294). These heterogeneity axes inform brain–computer interface (BCI) choices and clinical positioning: when motor imagery is reliable and corticospinal tract (CST) integrity is adequate on transcranial magnetic stimulation (TMS)–evoked motor potential (MEP) testing or diffusion tensor imaging (DTI) (295), intention-contingent motor-imagery electroencephalography (MI-EEG) BCIs can be paired with functional electrical stimulation (FES), robotic assistance, or virtual reality (VR) to deliver assistance at the moment of brain intent for selective motor control training (292). Where motor imagery is weak, fatigue is prominent, or speech/manual output is limited, P300 event-related potential (P300) or steady-state visual evoked potential (SSVEP) BCIs provide robust augmentative and alternative communication (AAC) or alternative feedback channels, with pragmatic dosing in short, feedback-rich bouts tailored to pediatric fatigue and attention profiles (291, 296).

#### 4.6.4 BCI-based rehabilitation in CP: evidence and outcomes

Building on these theoretical foundations, preliminary studies have begun to assess the feasibility of applying BCI technology to pediatric motor disorders. Currently, few studies have examined the efficacy of BCI technology-based cerebral palsy rehabilitation, but some have examined the ability of children with perinatal stroke to use BCI, for example, Z. Jadavji et al. (297) compared 21 children with perinatal stroke and 24 typically developing peers on two EEG-based BCI motor-intention tasks. Children with perinatal stroke achieved control performance comparable to controls (Cohen's *κ* > 0.40), indicating that they can reliably operate an EEG-based BCI. From a rehabilitation perspective, this provides preliminary evidence that pediatric motor-impairment populations can generate usable intention signals for BCI control, laying the groundwork for future BCI-supported motor rehabilitation in cerebral palsy. In addition, E. D. Floreani et al. (298) based user-centered design principles to develop a home BCI program for children with tetraplegic cerebral palsy, in which children and their families were involved in every stage of the software design process to evaluate and provide feedback on its use. Seven families have successfully and independently used the system at home, suggesting that the design and successful implementation of user-centered software for home use will both inform on the feasibility of BCI as a long-term access solution for children with neurological disabilities as well as decrease barriers of accessibility and availability of BCI technologies for end-users. Meanwhile, N. Usama et al. (299) showed that error-related potentials (ErrPs)—the brain's automatic response to perceived errors during a task—can be reliably detected in children with cerebral palsy, with classification performance around the mid-80% range. ErrP detection allows a BCI system to recognize when its output does not match the user's intention and to adjust or correct commands in real time. This is particularly relevant for children with CP, who frequently experience attentional lapses and delayed or imprecise motor feedback. A BCI that can automatically detect and correct such errors has the potential to reduce frustration, improve interaction efficiency and adherence, and ultimately make BCI-supported rehabilitation more tolerable and effective for this population.

#### 4.6.5 Limitations and future directions

Despite the potential application of BCI techniques for the rehabilitation of children with cerebral palsy, there are many gaps and shortcomings in the field. Future studies should tailor BCI paradigms to the developmental and neurophysiological characteristics of children with cerebral palsy (CP). Compared with adults, children exhibit broader yet less stable neural plasticity, producing variable EEG patterns that require adaptive algorithms capable of recalibration over time. Motor imagery ability is often limited—especially in spastic or dyskinetic subtypes—necessitating multimodal feedback (visual, auditory, proprioceptive) to strengthen motor representations. Attentional fluctuations and cognitive fatigue further hinder engagement, highlighting the value of gamified, reward-based interfaces to enhance motivation and adherence. Moreover, rehabilitation goals differ across subtypes, with spastic CP emphasizing tone regulation and ataxic CP focusing on coordination and timing. Because pediatric rehabilitation relies heavily on family participation, home-based, user-friendly BCI systems integrating caregiver-assisted protocols are essential for long-term clinical effectiveness. Conduct long-term follow-up studies to assess the durability and effectiveness of the BCI system in real-world applications. Develop more personalized and adaptive algorithms to accommodate the specific needs and abilities of children of different ages and conditions. To further explore the application of BCI technology in the clinical rehabilitation of children with cerebral palsy.

### 4.7 Neuropathic pain (NP)

#### 4.7.1 Disease presentation and standard of care

Neuropathic pain (NP) is a chronic pain condition resulting from lesions or dysfunction of the somatosensory nervous system, including disorders such as trigeminal neuralgia, diabetic polyneuropathy, postherpetic neuralgia, and post-stroke central pain. Patients frequently report persistent or intermittent spontaneous pain—burning, tingling, or electric-like sensations—often accompanied by sensory hypersensitivity and allodynia. Such chronic pain not only causes severe physical discomfort but also impairs sleep, emotional stability, and social functioning, leading to a profound reduction in quality of life (300). The overarching rehabilitation goals for NP are to alleviate pain, restore functional capacity, and improve psychosocial well-being, requiring an integrated approach that combines pharmacological, physical, and psychological interventions.

Current rehabilitation for NP relies on a multimodal and multidisciplinary framework, combining pharmacologic therapy (antidepressants, anticonvulsants, or opioids) with physiotherapy, occupational therapy, and cognitive-behavioral therapy (CBT). Physical interventions such as transcutaneous electrical nerve stimulation (TENS) and electrical muscle stimulation (EMS) are used to modulate sensory input and enhance functional reconditioning, while CBT targets maladaptive pain coping and catastrophizing (301–303). In more severe or medication-refractory cases, spinal cord stimulation (SCS) has become a cornerstone neuromodulatory therapy. SCS delivers low-voltage electrical currents to the dorsal columns of the spinal cord, modulating ascending pain transmission and restoring inhibitory control over hyperexcitable nociceptive pathways. This technique significantly reduces pain intensity and improves functional mobility. Recent advances in closed-loop SCS have further optimized outcomes by continuously monitoring spinal responses (e.g., evoked compound action potentials) and automatically adjusting stimulation intensity in real time (304, 305). There isn't a single “standard” duration for neuropathic-pain (NP) rehabilitation. Interdisciplinary pain-rehabilitation programs (IPRPs): intensive day-hospital or outpatient blocks commonly run 3–4 weeks daily, or 7–8 weeks at ∼3 days/week; broader outpatient programs often span ∼2–12 weeks depending on site and cadence (306, 307). Psychological therapy (CBT for chronic pain): most protocols use 8–12 sessions (≈6–12 weeks), 30–50 min each, with structured skills practice between sessions (308). Graded Motor Imagery/Mirror Therapy (commonly used for NP phenotypes like CRPS/phantom pain): typical blocks are ∼4–6 weeks. RCTs frequently use 4-week daily mirror-therapy schedules; staged GMI programs often progress about 2 weeks per phase (laterality → imagery → mirror), totaling ≈6 weeks, with home practice (309). Exercise-based rehabilitation: delivered in time-limited blocks (often 6–12 weeks) and then transitioned to ongoing home programs; authoritative guidance emphasizes establishing a long-term exercise routine to sustain gains in pain, function, mood, and QoL (310).

NP is most often a chronic, fluctuating condition in which complete and durable remission is uncommon; long-term burden on function and quality of life is typical (300). Prognosis varies by etiology: central NP after spinal cord injury frequently persists and is difficult to treat over time (311). Postherpetic neuralgia (PHN) tends to gradually remit in some patients, yet pain can persist for months to years, with persistence more likely in older adults (312). Painful diabetic peripheral neuropathy shows a variable natural history—some experience partial or even spontaneous improvement, but many remain chronically symptomatic (313). In complex regional pain syndrome (CRPS), many improve within 6–13 months, though a substantial minority develop long-term symptoms and disability beyond a year (314). Across NP conditions, risk of persistence is increased by older age and greater initial pain severity in PHN and by markers of ongoing nerve/central sensitization in other syndromes, underscoring heterogeneous but often prolonged trajectories (315).

#### 4.7.2 Role of neuroplasticity in the rehabilitation process

Maladaptive neuroplasticity in neuropathic pain strengthens nociceptive pathways, manifesting as central sensitization: synaptic “gain” increases within dorsal horn and related circuits, pain thresholds are lowered, responses are amplified, and receptive fields may expand beyond the original painful area (316). At the cortical level, regions such as primary somatosensory cortex (S1) undergo reorganization that is associated with pain persistence; when symptoms improve, this abnormal reorganization can recede toward a more typical pattern (317). Conversely, adaptive plasticity can be harnessed to reinforce more appropriate networks. Graded motor imagery and mirror therapy progressively engage systems underlying movement and body representation, reducing pain while recalibrating distorted cortical maps (318). Sensory discrimination training uses feedback-guided fine tactile tasks to refine somatosensory maps over the painful region with the same goal (319). In addition, modulation of the primary motor cortex (M1) can rebalance cortical excitation–inhibition and recruit descending inhibitory pathways, providing a top-down mechanism consistent with analgesia (320). Overall, the clinical trajectory of neuropathic pain reflects the balance between maladaptive sensitization and targeted recalibration; when practice or stimulation is aligned in frequency and timing with the intended networks, adaptive pathways are strengthened, maladaptive circuits are down-weighted, and long-standing pain becomes more controllable.

#### 4.7.3 BCI use in NP

Within this established rehabilitation framework, brain–computer interface (BCI) technology introduces a fundamentally different yet complementary mechanism of pain modulation. Traditional neuromodulation methods (e.g., TENS, SCS) act peripherally or spinally, indirectly influencing pain transmission (321, 322), whereas BCIs act centrally, decoding cortical activity related to pain perception or emotional distress. This allows BCIs to provide objective, real-time monitoring of pain states, which is particularly valuable given the subjective and fluctuating nature of chronic pain reports (323, 324). Moreover, BCIs can serve as adaptive controllers for existing modalities—by integrating EEG or ECoG pain biomarkers to automatically regulate the intensity or pattern of TENS or SCS stimulation. This central–peripheral integration transforms passive stimulation into a closed-loop, patient-specific therapy that continuously aligns intervention strength with the patient's actual neural state (325). Equally important, BCIs can support self-regulation and neurofeedback training, enabling patients to consciously modulate cortical pain networks and emotional reactivity (326). This represents a key divergence from conventional passive therapies: BCIs actively engage patients in the modulation process, enhancing motivation, sense of control, and long-term adherence—factors strongly associated with sustained pain reduction and improved quality of life. Widely used devices primarily deliver programmed neuromodulation or peripheral stimulation for symptom control—for example, spinal cord stimulation (SCS), including 10-kHz high-frequency SCS, which improved outcomes in refractory painful diabetic neuropathy in a randomized trial; dorsal root ganglion (DRG) stimulation, which outperformed conventional SCS for complex regional pain syndrome in the ACCURATE RCT; and transcutaneous electrical nerve stimulation (TENS) and primary-motor-cortex rTMS, which have mixed or condition-specific evidence and do not decode brain state in real time (327).

Decoding-based BCI used in chronic neuropathic pain—particularly EEG or real-time fMRI neurofeedback—can improve quality of life (QoL) by enabling patients to self-modulate pain-related brain activity, thereby reducing pain intensity and pain interference (328). Patients have learned to control activation in the rostral anterior cingulate cortex with real-time fMRI, producing concurrent reductions in experimentally evoked or chronic pain, a mechanism that links brain-state control to symptom relief (329). Consistent effects are also reported with EEG neurofeedback for central NP after spinal cord injury, including self-managed, home-based protocols that reduce pain and are feasible for longer-term use—features that support participation and day-to-day function, core contributors to QoL (330).

In NP, sensorimotor involvement varies by lesion site/extent and time since onset: peripheral and dorsal-root lesions produce length-dependent sensory loss and hyperalgesia, whereas central lesions after spinal cord injury are linked to persistent pain with primary somatosensory cortex (S1) reorganization whose degree correlates with ongoing pain; across NP etiologies, central sensitization—an LTP-like increase in dorsal-horn synaptic efficacy—lowers thresholds, amplifies responses, and can expand receptive fields; symptoms often fluctuate diurnally under circadian control (331). These axes shape brain–computer interface (BCI) choices and clinical positioning. When a cortical pain-modulatory target is identifiable and patients can engage in imaging-guided practice, real-time functional MRI (rt-fMRI) neurofeedback targeting the rostral anterior cingulate cortex provides an NP-specific route to self-regulation of pain intensity (329). For cohorts with limited mobility or access—notably central NP after spinal cord injury—home-based electroencephalography (EEG) neurofeedback BCIs offer a practical alternative, with short, feedback-rich sessions that fit fatigue and attention limits and are now being deployed and evaluated in SCI-NP populations.

#### 4.7.4 BCI-based rehabilitation in NP: evidence and outcomes

Recent pilot and feasibility studies indicate that BCIs may play a viable role in neuropathic-pain management, complementing conventional neuromodulation and rehabilitation approaches. For example, G. V. Aurucci et al. (332) proposed a BCI–VR–TENS system that decodes EEG and skin conductance signals associated with neuropathic pain in real time and automatically delivers multisensory stimulation via virtual reality and transcutaneous electrical nerve stimulation. This system achieved approximately 75% online pain-detection accuracy and resulted in a 50% reduction in Neuropathic Pain Symptom Inventory (NPSI) scores. Although lack of a control group, this study provides compelling proof-of-concept evidence that BCI-based systems can actively monitor and modulate pain rather than passively deliver stimulation. Beyond direct neuromodulation, M. Sun et al. (333) proposed a BCI-based music therapy for feedback-driven emotion regulation. EEG signals were used as emotional control inputs to generate adaptive music stimuli, which promoted hormonal balance, reduced negative affect, and supported self-regulation in patients with mood disturbances related to chronic pain. This approach highlights the potential of BCI to target the affective dimension of pain, integrating emotional and physiological modulation within the same neurofeedback framework. Demarest et al. (326) tested a neurofeedback BCI in which patients with chronic upper-extremity pain learned to up-regulate a pain-modulatory theta rhythm while receiving contingent vibrotactile feedback. Participants who successfully increased this cortical activity experienced meaningful reductions in both pain severity and pain interference, suggesting that training specific brain rhythms can provide a non-pharmacological route to pain control that targets central mechanisms rather than peripheral symptoms alone, it illustrates how BCI-based neurofeedback can be integrated into chronic pain management as an active, self-regulation–focused adjunct to standard care. Expanding this approach to home-based systems, Al-Taleb et al. (221) conducted a feasibility study showing that patients with central neuropathic pain after spinal cord injury could independently use a portable EEG–BCI platform at home and achieve measurable pain relief. Sakel et al. (334)implemented an 8-week family-assisted EEG neurofeedback program for individuals with chronic neuropathic pain, reporting significant reductions in pain intensity, depressive symptoms, and catastrophizing scores. These findings suggest that accessible, user-friendly BCIs may enhance patient autonomy by integrating pain self-management into everyday life.

#### 4.7.5 Limitations and future directions

These studies introduce a novel method for treating neuropathic pain using BCI technology for real-time pain monitoring and intervention, showcasing its feasibility and effectiveness. However, patient responses to VR and TENS can vary, and individual differences have not been thoroughly examined. Additionally, music therapy's success may rely heavily on personal musical preferences and cultural background, potentially reducing its effectiveness for those indifferent to music.

## 4 Conclusion

BCI technology shows great promise in neurological rehabilitation, especially with non-invasive methods for treating nervous system disorders. Neurological disease benefit with a common mechanism: closed-loop decoding of intention plus salient feedback to drive neuroplasticity. In stroke, MI-BCI paired with FES/VR/soft robotics improves upper-limb, hand, and gait outcomes beyond conventional therapy and is expanding to cognition and mood. In MS, early studies demonstrate feasibility and gait gains with BCI-FES, but controlled trials and broader QoL endpoints are sparse. In ALS, invasive speech BCIs restore fast, durable communication for some patients and home use is feasible but performance in complete locked-in state remains a gap. For SCI, intracortical/epidural “brain-spine” links and BCI-triggered FES/exoskeletons enable purposeful reaching and over-ground walking, More and more evidence indicates that it can also be used in a family environment. In PD, BCIs reliably detect motor intent and show promise for reducing tremor/rigidity and enabling MI-BCI training. In CP, children (including perinatal stroke) can operate BCIs, and user-centered home systems are practical, despite evidence remains preliminary. For neuropathic pain, real-time BCI detection coupled to VR/TENS or affect-aware music feedback reduces pain and improves regulation. All in all, significant progress has been made in integrating BCI with other rehabilitation devices. Current research focuses on non-invasive BCI, its integration with equipment, using high-level cognitive signals, and applying deep learning for signal processing and pattern recognition. Currently, the main limitations of research in the field of brain-computer interface technology for neurological disease rehabilitation include small samples, heterogeneous protocols, limited long-term data, variable signal quality after injury, and insufficient personalization and usability work.

## 5 Perspective

Despite existing challenges, brain-computer interface (BCI) technology holds significant promise for neurological rehabilitation. Advances in science, technology, and interdisciplinary collaboration could enable BCIs to transform therapeutic approaches and enhance patients’ quality of life. Future research should focus on developing an integrated pipeline:

Personalized Cortical Network Modeling: Construct individualized, continuously updated cortical-network simulators using EEG and MRI/fMRI data to model patient-specific brain dynamics. These models would allow in silico pre-testing of candidate therapeutic tasks, training intensities, and stimulation parameters to optimize clinical intervention plans.

Self-Architecting Decoders: Develop decoders capable of autonomously assembling optimal signal processing chains (filters, feature extractors, classifiers) from data. These decoders should self-recalibrate to maintain performance despite signal degradation or electrode drift.

Adaptive Therapy Protocols: Implement therapy regimens that dynamically adjust task difficulty and reward schedules based on real-time neural learning metrics. Modalities (e.g., transitioning from pure motor imagery to haptic-augmented motor imagery) should be adaptively switched to overcome plateaus in neuroplasticity.

Integrated Sensory Feedback: Combine BCI with peripheral nerve interfaces and targeted vibrotactile or optogenetically-inspired stimulation to provide proprioceptive and tactile feedback. This “writing back” of sensation to impaired neural circuits aims to restore the perception of limb movement and contact, facilitating motor relearning.

This integrated system enhances multiple aspects of rehabilitation: optimized intervention selection, improved model robustness, sustained learning motivation, and biologically relevant sensory feedback. Consequently, it has the potential to promote more efficient, reliable, and sustainable functional recovery. Critically, as BCI technology advances, prioritizing patient welfare and adhering to rigorous ethical standards remains paramount (335).
